# A system for monitoring the functional status of older adults in daily life

**DOI:** 10.1038/s41598-023-39483-x

**Published:** 2023-07-31

**Authors:** Björn Friedrich, Lena Elgert, Daniel Eckhoff, Jürgen Martin Bauer, Andreas Hein

**Affiliations:** 1 Department of Health Services Research, Carl von Ossietzky University, Ammerländer Heerstraße 114-118, 26129 Oldenburg, Lower-Saxony Germany; 2 Peter L. Reichertz Institute for Medical Informatics of TU Braunschweig and Hanover Medical School, Karl-Wiechert-Allee 3, 30625 Hanover, Lower-Saxony Germany; 3 School of Creative Media, City University of Hong Kong, Tat Chee Avenue, Kowloon, Hong Kong China; 4 Geriatric Centre of the Heidelberg University, University of Heidelberg, Rohrbacher Straße 149, 69126 Heidelberg, Baden–Wuerttemberg Germany

**Keywords:** Preclinical research, Translational research, Rehabilitation

## Abstract

Functional decline in older adults can lead to an increased need of assistance or even moving to a nursing home. Utilising home automation, power and wearable sensors, our system continuously keeps track of the functional status of older adults through monitoring their daily life and allows health care professionals to create individualised rehabilitation programmes based on the changes in the older adult’s functional capacity and performance in daily life. The system uses the taxonomy of the International Classification of Functioning, Disability and Health (ICF) by the World Health Organization (WHO). It links sensor data to five ICF items from three ICF categories and measures their change over time. We collected data from 20 (pre-)frail older adults (aged $\ge$ 75 years) during a 10-month observational randomised pilot intervention study. The system successfully passed the first pre-clinical validation step on the real-world data of the OTAGO study. Furthermore, an initial test with a medical professional showed that the system is intuitive and can be used to design personalised rehabilitation measures. Since this research is in an early stage further clinical studies are needed to fully validate the system.

## Introduction

Older adults are prone to functional decline, this leads to an increased need of care and eventually, they might need to move to a nursing home. Moving to a nursing home has a negative effect on the three basic psychological needs competence, relatedness, and autonomy^1^. This also puts a financial burden on the older adult, relatives, and the health care system. In addition, there is a lack of medical professionals which affects the quality of care^2^. This issue becomes increasingly serious, as many nations are undergoing a demographic change^3^. One solution to face these challenges might be to enable older adults to live longer independent lives in their own domestic environments or in other words enable them to age in place.

For that purpose, we introduce a new Older Adults Monitoring System (OdAMS) that monitors the functional status of older adults in daily life and enables medical professionals to create individualised rehabilitation programmes that have a better outcome than generic programmes^4^. This helps older adults to maintain their functional abilities and live an independent life. OdAMS can also be used by medical professionals to monitor the effect of rehabilitation programmes. One of the benefits is the measurement in daily life without creating a testing situation. Real life mobility measurements tend to be better indicators and predictors than test scores, e.g. real life gait speed is a good predictor for the duration of hospitalisation and mortality^5–8^. Many factors affect the health and functional status of older adults and that makes each individual an unique case^9,10^. This fact is well-known in geriatrics medicine and was found by analysing the sensor data from older adults collected in their domestic environments in daily life^11^. OdAMS was specifically tailored to the individual by being adapted using data from the individual only. The data basis was formed by inertial measurement units (IMU), home automation, and power consumption sensor data. Since the data was collected in one of the most private spaces of older adults, unobtrusive privacy preserving sensors had to be used. Moreover, this type of sensor is accepted by older adults amongst different cultures, especially for the purpose of keeping their autonomy^9–14^. Three components linked the sensor data to the ICF categories: Activities and Participation (items: d450, d460), Body Functions (item: b530), and Environmental Factors (items: e115, e245)^15^. The ICF items were taken from the ICF Core Set: Geriatrics Comprehensive^16^.

## State of the art

Using smart-home and wearable sensors in the medical context of older adults has come into focus of researchers. Much effort was given to conduct studies to get insights in the impact of such system and their benefit for measuring health indicators^17–21^. One main goal was activity and behaviour monitoring for the purpose of drawing conclusions on the health status^22–24^. While activity monitoring is unspecific, other researchers investigated the detection of serious specific incidents like fall detection^25–27^. A few attempts to measure the functional decline of older adults in daily life have been made. All of them are focusing on selected specific aspects^19,24,28–30^. However, focusing on one aspect does not give a holistic view on the older adult and neglects the complexity of the interaction between different aspects like physical functional decline and mental functional decline. Moreover, previous research does not link their outcomes and model outputs to the ICF and that may create a barrier for medical professionals and physician–researchers to use the results effectively. OdAMS addresses both disadvantages of the current state of the art. A comprehensive state of the art analysis for each sub-component can be found in the related articles^31–34^.

## Methods

The foundation of OdAMS is the data collected during the observational randomised pilot intervention study OTAGO^35^. The system was comprised of three components and used the ICF as output. In the this section we describe the study design, the system, and all of its parts. The data of the participants and the source code of the system can be found in the supplementary materials??.

The OTAGO study was conducted according to the guidelines of the Declaration of Helsinki, and approved by the Institutional Review Board of Carl von Ossietzky University (protocol code: Drs.27/2014, date of approval: 30.04.2014).

### Study design

The aim of the study was to investigate the “Effect of the fall prevention intervention program OTAGO on older persons with frailty and prefrailty—an analysis with conventional geriatric and technology–based assessments”. The observational randomised pilot intervention study was conducted from 07/2014 to 06/2015. Data of 20 participants (17 female, 3 male) was collected during 36.5 weeks on average. Two participants passed away during the study. Considering the pilot study character and technology focused approach, the number of participants was sufficient. The inclusion criteria for the study were

1. Age $$\ge$$ 75
2. At least pre–frail by the definition of Fried^36^
3. Living alone within the city limits of Oldenburg
4. Able to move inside the flat as needed

and the exclusion criteria were

1. Not able to move inside the flat as needed
2. Keeping pets that move freely inside the flat
3. Living with other people
4. Strong visual impairment
5. Contraindication against the OTAGO exercise programme
6. Unable to understand the purpose of the study and the study itself

The participants were randomly assigned to the control group and intervention group. During the study every 4 weeks (avg. 31.3 days, SD: 5.3 days) a battery of geriatrics assessments was executed and physical parameters were measured in the homes of the older adults by a licensed physiotherapist. The assessments used for this research are Hand Grip Strength (HGS), Frailty Index (FI)^36^, Short Physical Performance Battery (SPPB)^37^, Timed Up & Go (TUG)^38^, instrumental Activities of Daily Living (iADL)^39^, and the physical parameter body weight. Tables 1 and 2 are showing the characteristics of the study cohort at baseline (T0) and the end of the study (T10) (edited^31^).

**Figure 1:**
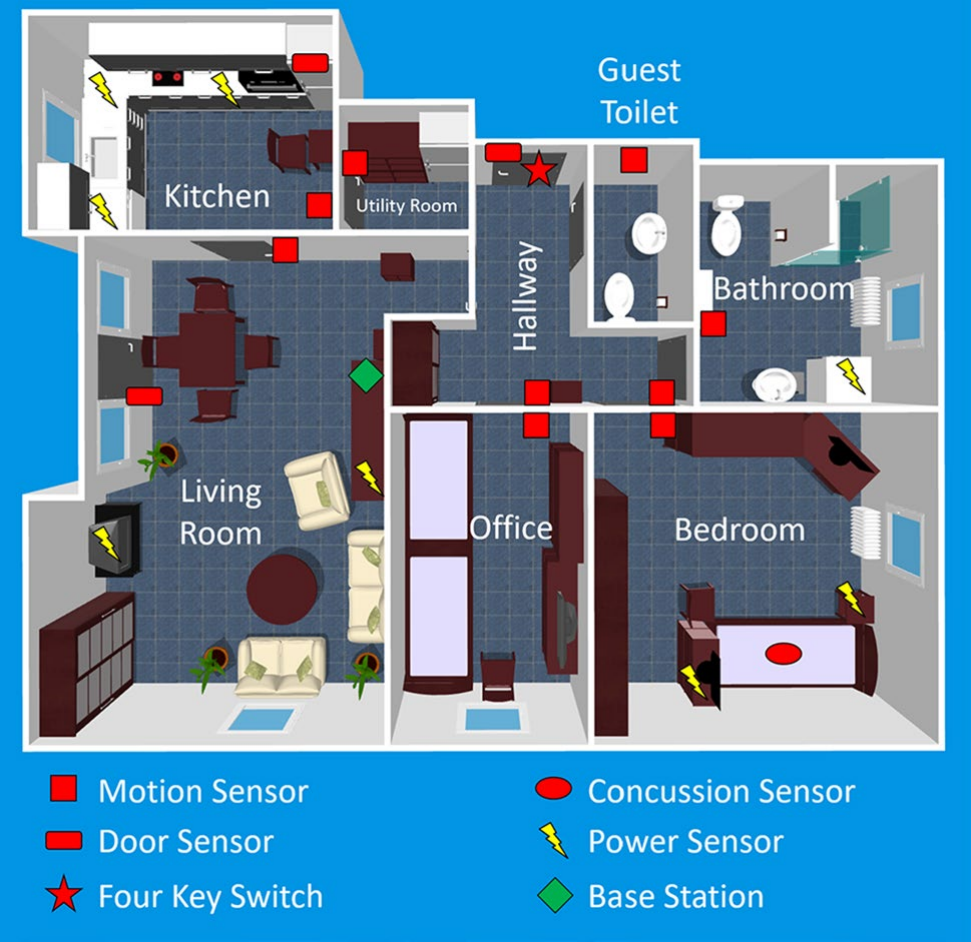
An illustration of one of the flats, which was equipped with the sensor system.

The sensor system, which was installed in the homes of the participants, was comprised of home automation, power consumption, and two wearable sensors—one IMU and one GPS receiver. The power consumption sensors were installed between appliances and outlets, measuring the electricity consumption of the connected device. In terms of home automation sensors, passive infrared (PIR) motion sensors and door contact sensors were installed in each apartment. The door contact sensors were installed at the front door, windows, fridge, and the backdoor, if applicable. Additionally, a concussion sensor was placed in-between the slatted frame and the mattress. An extra four-key switch was placed near the front door and the participants were asked to turn the switch when another person enters or leaves the flat. All sensors send transmitted their data to a base station using a wireless connection. An example of a flat is shown in Fig. 1. Since each participant was free to choose the sensors, some sensor setups were different.

**Table 1:** Characteristics of the study cohort at baseline (T0) (edited^31^).

n=20, m=3, f=17	Age (years)	Body weight (kg) m/f	HGS (kg) m/f	FI (pts.)	SPPB (pts.)	TUG (s)	iADL (pts.)
Mean	84.8	66.2/69.2	17.1/14.1	1.9	6.0	17.9	7.3
SD (±)	5.2	3.6/17.3	5.1/7.0	0.7	2.3	5.3	1.4
Range (min–max)	76.0–92.0	61.9/43.8–70.8/115.9	11.7/3.7–24.0/33.0	1.0–3.0	3.0–11.0	11.2–31.6	3.0–8.0

**Table 2:** Characteristics of the study cohort at the end (T10) (edited^31^).

n=28, m=3, f=15	Age (years)	Body weight (kg) m/f	HGS (kg) m/f	FI (pts.)	SPPB (pts.)	TUG (s)	iADL (pts.)
Mean	84.5	66.1/71.0	16.8/12.2	2.0	6.6	16.4	6.1
SD (±)	4.9	5.4/19.1	4.2/3.7	1.0	2.9	6.0	2.3
Range (min–max)	77.0–93.0	59.1/42.7–72.1/123.7	13.3/5.0–22.7/18.0	0.0–4.0	2.0–12.0	8.5–30.1	1.0–8.0

### International classification of functioning, disability and health

**Figure 2:**
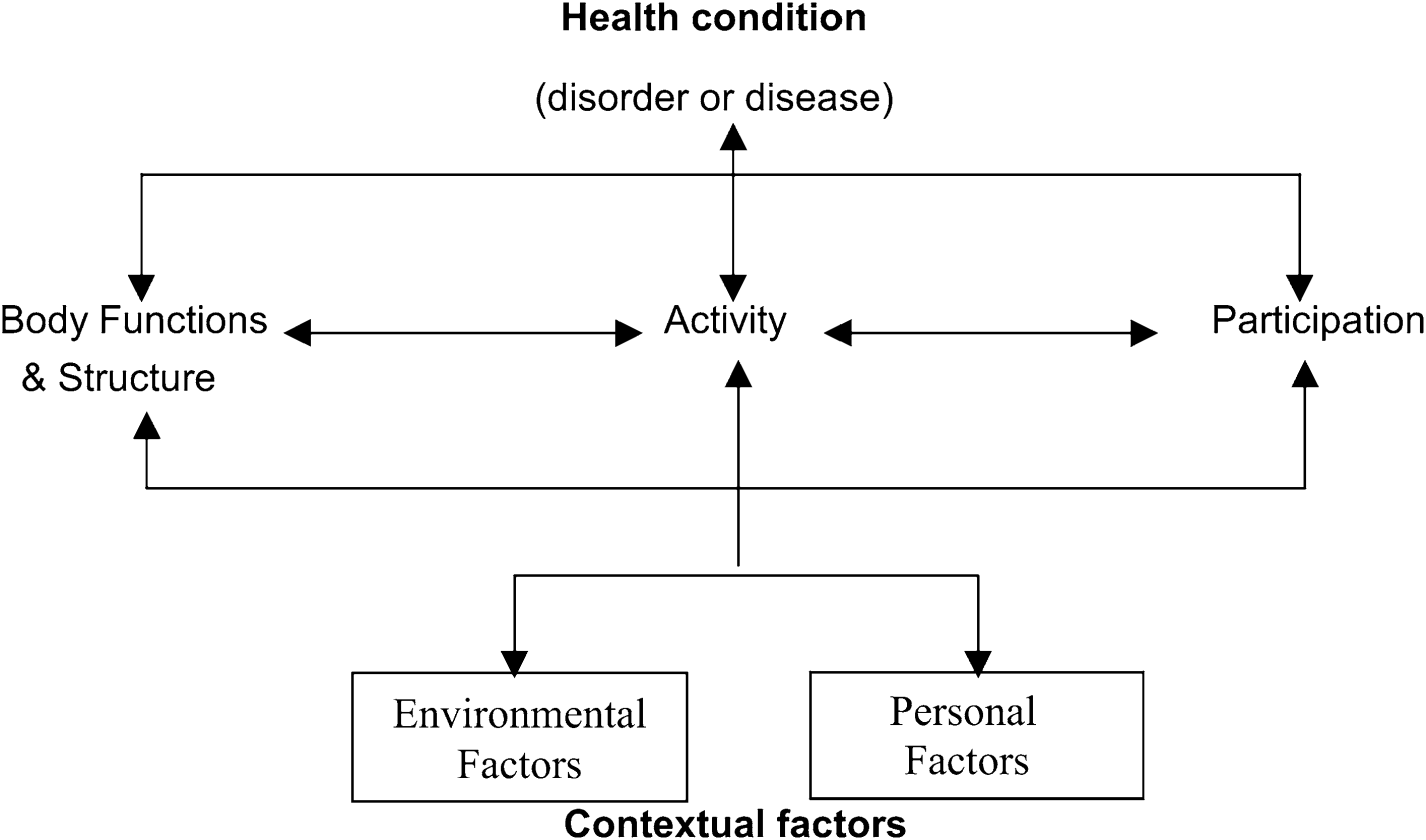
The disease model used as foundation of the ICF^15^.

The ICF was developed by the WHO in 1980 and received approval from all member states in 2001. The main purpose was to establish a common standard for describing health and health-related states from individual scale to population scale. The benefit for patients is that not only the diagnoses are covered but also the physical, mental, and social aspects of the health condition^15^. Figure 2 illustrates the ICF’s basic model of disability^15^. The holistic view on the patient’s condition enables medical professionals to plan rehabilitation programmes considering more aspects than the diagnosis only. The ICF is comprised of too many items to be applied directly. Therefore, the ICF Core Sets were introduced as reference framework^16^.

### System

**Figure 3:**
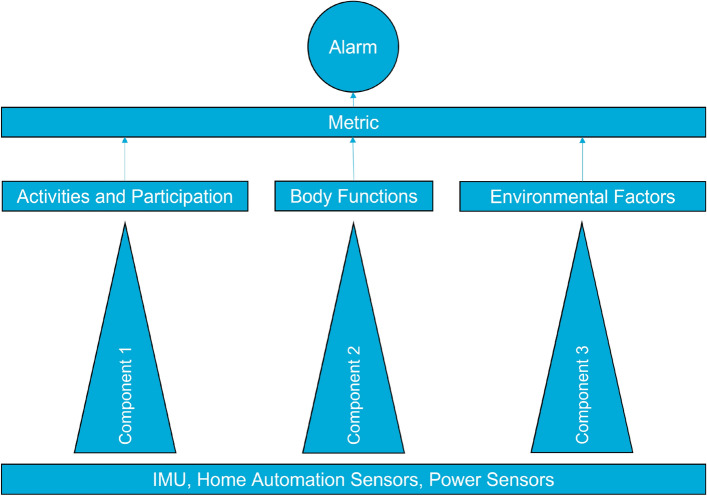
The final system comprised of three components.

The final system is illustrated in Fig. 3. Each component was linking data to one ICF category and one or two ICF items. The output of the first component monitored the assessment scores of standardised geriatrics assessments and the changes using IMU data. The second component monitored the change in body weight using home automation and power consumption sensors. The third components also used home automation and power consumption sensors but monitored the utilisation of electric appliances and the activity. The late fusion in the metric was done by logical or conjunction of the outputs. A change in one of the categories triggered an alarm and a medical professional could track the reason down to the ICF item and take the necessary measures. The metric and the components are described in the following subsections.

### Metric

All three categories were considered as equally important and the metric was defined accordingly. The metric’s input was the outputs $C_1$, $C_2$, $C_3$ of the three components and the outputs were concatenated by logical or $(\vee )$. If one of the inputs was 1, i.e. one component was indicating a change an alarm was raised. No change was indicated by 0 by the components. Equation (1) shows the metric as Boolean algebraic expression.

1 $$\begin{aligned} C_1\vee C_2 \vee C_3 \end{aligned}$$

### Component 1: activities and participation

The first component was comprised of two deep neural networks. The networks took the magnitude, i.e. Euclidean Norm of all three axis, of the IMU sensor’s low-range accelerometer, wide-range accelerometer, gyroscope, and magnetometer as input and predicted the SPPB and the TUG scores, not the underlying raw values (e.g. seconds in case of the TUG), the older adult would achieve. The deep neural network predicting the SPPB score was linking the IMU data to the ICF item d460: Moving around different locations and the deep neural network predicting the TUG score is linking to d450: Walking. Table 3 shows the association of the assessment and ICF scores. When one of the two deep neural networks detected a change, e.g. TUG score from 1 to 2 and so from ICF score 0 to 2, a 1 would be propagated to the metric function. When no change was detected a 0 would be propagated. The component also provided the information if the change was caused by an improvement or deterioration. The component is described in greater detail in reference^31^.

**Table 3:** The linking of the assessment scores to the ICF scores.

Type/level of impairment	No	Mild	Moderate	Severe	Complete
SPPB score	12, 11, 10	9, 8, 7	6, 5, 4	3, 2, 1	0
TUG score	1	–	2, 3	–	4
ICF score	0	1	2	3	4

### Component 2: body functions

Component 2 measured the change in body weight by computing the time spent for preparing meals. The time was computed using the home automation and power consumption sensors attached to appliances in the kitchen. The meal preparation time showed a strong significant Spearman correlation of at least $\vert 0.99\vert$ with body weight and HGS. Moreover, the model could detect weight changes of 5% in 3 months as an indicator for malnutrition in older adults^40^. It was not possible to draw inferences from the algebraic sign of the correlation coefficient about if the participant gained or lost body weight and the magnitude of the body weight change. Therefore, the component’s output was 1 in case the body weight changed and 0 otherwise. Changes smaller than 1 kg were considered as normal fluctuations and were discarded during the component’s built process. The result was linked to the ICF item b530: Weight maintenance functions. The component was not available for participants who were not preparing meals themselves at home. The component is addressed in depth in reference^32^.

### Component 3: environmental factors

**Figure 4:**
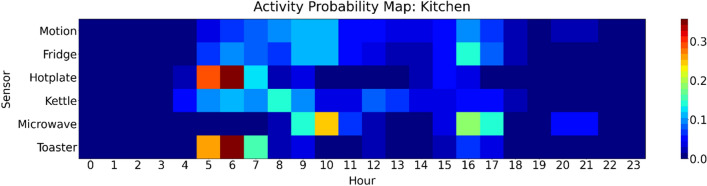
Activity probability map of participant 1’s kitchen for day 1^34^.

The third component was comprised of an unsupervised concept drift detection machine learning model and a mathematical model. The unsupervised concept drift detection model detected normal and abnormal days. The features of the model were activity probability maps built from home automation and power consumption sensors. One map contained the probability a sensor events occurs in a certain hour of the day. The average probability map of 7 days was used as baseline. The machine learning model evaluated the probability map of a new day against the baseline. If the probability that the map of the new day was from the same probability distribution was smaller than a given threshold, the day was considered as normal and abnormal otherwise. The baseline was updated with each normal day to adapt to the behavior of the person. Figure 4 shows an example activity probability map^34^. Since the activity probability maps included without limitation the use of appliances and devices in the apartment and the output was linked to e115: Products and technology for personal use in daily life.

The mathematical model was a weighted directed graph representing the motion sensors in the apartment of the older adult. Each vertex of the graph represented one room of the apartment and the edges where the possible transitions between the rooms are. The weights were the transition counts and used as activity measure. Every day was compared to the baseline and the difference in percent was computed. Two linear functions were fitted to the differences. The first linear function to the values of the control group and the second linear function to the values of the intervention group. The equations of the fitted lines are shown in Eqs. (2) and (3).

2 $$\begin{aligned} f_{\text {control}}(x)= & {} -\,8.5829\times 10^{-4}x+0.1340 \end{aligned}$$

3 $$\begin{aligned} f_{\text {intervention}}(x)= & {} -\,4.9948\times 10^{-5}x-1.0610\times 10^{-2} \end{aligned}$$

where *x* is a period in days counted from the beginning of the study (T0). The results showed that the activity of the intervention group was decreasing slightly, whereas the activity of the control group was decreasing stronger by a factor of 10. The result of the sensor graph was linked to the ICF item e245: Time–related changes.

The outputs of both models were combined by the following equation

4 $$\begin{aligned} f_{\text {env}}(x) = \vert s\vert + \frac{c}{p} \end{aligned}$$

where *s* is the slope factor of equations (2) and (3), *c* the abnormal day count, and *p* the time period T to T+1 the abnormal days were counted in. If $f_{\text {env}}$ was greater than a given threshold, component 3’s output was 1. For this research 0.3 was used. In clinical practice the threshold would be adjusted online based on the number of false positive alarms. The slope factor was chosen so, that the component was more sensitive towards a change for people not following any exercise programme. If no information were available *s* was set to 0.

The result could not be directly mapped to an ICF score and the output was indicating a change. A detailed description of the mathematical model for measuring e245 was published in^33^ and the unsupervised concept drift detection machine learning model in^34^.

### Ethics approval

The methods were performed in accordance with relevant guidelines and regulations and approved by Institutional Review Board of the Carl von Ossietzky University. (protocol code: Drs.27/2014, date of approval: 30.04.2014).

### Consent to participate

Written informed consent was obtained from all subjects involved in the study.

## Results

**Figure 5:**
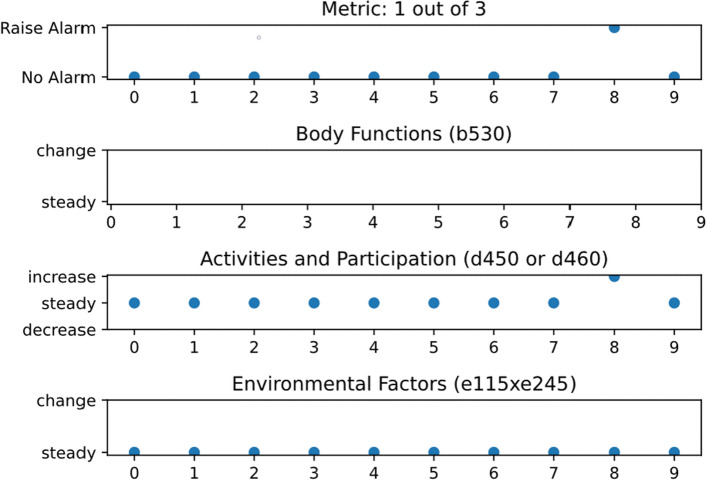
The system outputs of Mr. Mueller for month 0–9.

**Figure 6:**
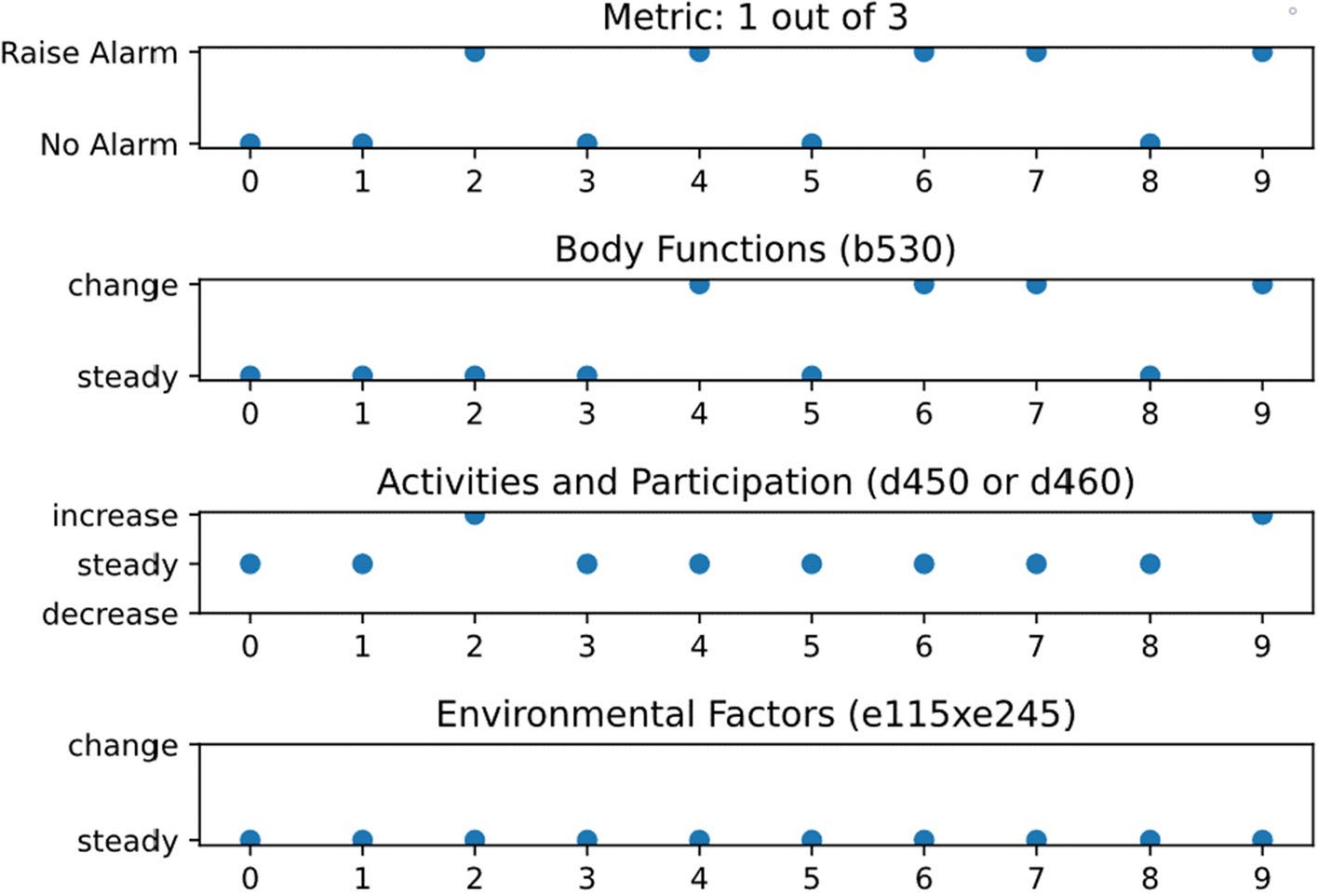
The system outputs of Mrs. Winter for month 0–9.

This section is comprised of two parts. In the first part the general results are explained and in the second part the two study participants “Mr. Mueller” and “Mrs. Winter”, whose names were changed due to privacy reasons, are examined in more detail and the application in physiotherapy is illustrated. The illustration is complemented by Figs. 5 and 6 showing the output of the system. The outputs of the remaining 18 participants can be found in the supplementary materials 6. The system raised an alarm every time a change occurred, even though not all components were available for each participant. Components 1 and 3 were available for each participant. Component 2 was not available for participants Mr. Mueller, 3, 4, 7, 9, 10, 12, 13, 14, and 19. These eight participants did not prepare meals themselves or had assistance. Table 4 shows the available ICF items, i.e. components for each participant. We used the assessment results to compensate missing IMU data in component 1 and still had missing values for participants 1, 7, 10, 11, and 15. The values were missing, because the assessment could not be performed due to their medical condition. Participant 14 was a special case, because the participant got cancer, underwent chemotherapy, and passed away. For component 3 there were only participant 7, and 14 with missing values. Participant 7 had a missing value in month 6. The participant was a special case as well, because he or she was hospitalised and moved to a nursing home after being discharge in month 6. Participant 14 had a missing value in month 2 due to medical condition. The cases of Mr. Mueller and Ms. Winter are described below as examples of how the system could be used by a licensed physical therapist to monitor the effect of an exercise programme to improve mobility and reduce the risk of falling.

Mr. Mueller was an 84 years old widower who sufferd from arterial hypertension, hypertensive heart disease, and subdural hemorrhage caused by a severe fall. He was living independently and was regularly supported by his children. The support included weekly grocery shopping, as well as transportation to medical appointments, but beyond that, he had no other contacts. Almost every day, one of the two children personally checked on their father. Mr. Mueller was pre-frail (3 pts. according to Fried’s Frailty Scale)^36^. At the beginning of the study, he scored 3 in the SPPB mobility test as well as in the TUG with 31.63 s^1,38^. Thus, he was considered at risk of falling and fall prevention training was advised. Following the OTAGO fall prevention exercise programme, Mr. Mueller received weekly home visits for the first two months and monthly visits thereafter. During these visits, he learned strength and balance exercises and received information about their importance for his everyday life. In addition to the exercises, Mr. Mueller was asked to take daily walks. Walking has never been Mr. Mueller’s favourite activity. Therefore, he seldom took any in the last few years. In the beginning, he managed to walk with his walking stick for 5 min to the corner of the house. After 10 months he was able to walk 30 min with his walking stick. As can be seen in Figure 1, there was no improvement or deterioration that caused an alarm up to month 8. The physiotherapist thought that this reflected Mr. Mueller’s regular routines with weekly shopping, recurring household activities, visits by the children, and fall prevention exercises. Slowly increasing the exercise programme’s intensity seemed to maintain functionality rather than significantly decreasing or increasing it. This was also evident in the mobility assessments, which were closely linked to the Activity and Participation component. In the SPPB, Mr. Mueller increased from 3 to 4 pts. over time and in the TUG he remained in category 3 but with an improvement from 31.63 to 22.31 s. In the third month, after cataract surgery, Mr. Mueller did not take any walks and fall prevention exercises for 2.5 weeks. However, this had no effect on the metric as can be seen in Fig. 5. The Body Functions component was deactivated for Mr. Mueller, because he had irregular lunches with his daughter. In months 8, the alarm indicated an increase in the ICF category Activities and Participation. The increase could also be seen in an improvement in TUG from 24.72 to 21.87 s. This improvement was relevant as it was above the Minimal Detectable Change (MDC) of 2.08 s^41^. In the SPPB, a re-improvement from 2 to 3 pts. points could be seen. The physiotherapist evaluated the changes as positive and increased the exercise programme’s intensity by increasing the walking time and adjusting the weights. Since the study ended no additional measures were taken. In a real-world case, the physiotherapist would tell Mr. Mueller to continue the exercises for the next 3 months without supervision and call if problems arise. After the 3rd month a follow-up examination would be done. However, since Mr. Mueller was not very motivated he might give less effort without supervision.

Mrs. Winter (Fig. 6) was a 76 years old women with arterial hypertension and arthrosis in hips and knees who was living in assisted-living scheme. She was self-sufficient except for daily lunch at the facility but she prepared all other meals by herself. In the beginning of the fall prevention training, she scored 5 pts. in the SPPB and 3 pts. in TUG with 24.06 s. Over the 10 months, she improved and ended the fall prevention programme with 10 pts. in the SPPB and in category 2 in the TUG with 11.90 s. In months 2, 4, 6, 7, and 9 the system alerted the physiotherapist. The body functions component indicated a change in months 4, 6, and 7. However, checking the weight with Mrs. Winter and asking about self-care revealed no abnormalities and was identified as normal fluctuations by a nutritionist. Alarmed by the metric, the physiotherapist noted an increase in the Activities and Participation component in month 2 and month 9. In month 2, the SPPB score changed from 4 to 6 pts. And in month 9 the SPPB score changed from 8 to 9 pts. The TUG remained in category 2 but increased from 15.40 to 13.12 s. The increase indicated that the right time had come for the physiotherapist to adjust the programme. The walking time could not be increased since Mrs. Winter was already taking long walks for 60 min. The intensity was raised by increasing the weight during strength exercises. After the 10 months, the physiotherapist ended the fall prevention training with Mrs. Winter. In a real-world case the physiotherapist would suggest that Mrs. Winter should continue the exercises on the current intensity level on her own and calls if problems arise. A follow-up in about 6 weeks would be done.

**Table 4:** The available components, i.e. ICF items, for each participant.

ID	ICF items
Mr. Mueller	d450, d460, e115
Mrs. Winter	d450, d460, b530, e115, e245
1	d450, d460, b530, e115, e245
2	d450, d460, b530, e115, e245
3	d450, d460, e115, e245
4	d450, d460, e115, e245
5	d450, d460, b530, e115, e245
6	d450, d460, b530, e115, e245
7	d450, d460, e115
8	d450, d460, b530, e115, e245
9	d450, d460, e115, e245
10	d450, d460, e115, e245
11	d450, d460, b530, e115
12	d450, d460, e115, e245
13	d450, d460, e115, e245
14	d450, d460, e115
15	d450, d460, b530, e115, e245
16	d450, d460, b530, e115, e245
17	d450, d460, e115, e245
18	d450, d460, b530, e115, e245

## Discussion

OdAMS proved its usefulness for creating individualised rehabilitation programmes. The results showed that the component 1 for Activities and Participation was the most accurate one and delivered the most detailed information. Moreover, analysing the metric showed that none of the components was redundant. There were alarms triggered by a single component only. With privacy in mind, it would have been good to identify redundant components and remove them according to the data economy principle. In addition, it is important that all data is stored and processed on a computer in the flat of the older adult. Only the alarm should be sent to a server via the internet. Even though, the system’s computing complexity was ${\mathcal {O}}\left( n^2\right)$ and dominated by the machine learning algorithms and the matrix multiplications inside them, OdAMS could be run on an ordinary computer. However, that might change in the future if the system is extended by new components. There are a few issues that must be addressed before the system can be used in clinical practice.

One issue is that the data was collected during pre-clinical pilot intervention study and the cohort size was small. Therefore, the components must be evaluated in separate clinical studies designed for each specific purpose. At this stage of the research, it is not possible to draw conclusions and the results can only be considered indicative. Another point is that component 1 also needs some adjustments. The machine learning models processed intervals of 1s length. A new metric, e.g. a majority vote ensemble, could be implemented to output a single score over a certain interval, because a change in mobility in a 1s interval would be too sensitive and thus trigger too many alarms and is not meaningful. The shortest interval is 1s and the longest has infinite length. However, infinite length is more of theoretical interest. The interval can be chosen according to the opinion of a physiotherapist, e.g. if high sensitivity is needed a short interval could be chosen. Also, the machine learning models must be fine-tuned to the individual. In contrast to component 1, the components 2 and 3 detected changes instead of absolute ICF item scores. Component 2 was linked to Weight maintenance functions and detected changes instead of an absolute score of the item. The component neither gives the information if the older adult gained or lost weight. Moreover, the component was applicable for only a few participants; consequently, the findings must be very carefully interpreted and are of preliminary nature. Component 3 was detecting changes of the item e115: Products and technology for personal use in daily life. The scoring interval was + 4 to 4 from complete facilitator to complete barrier, but concluding a score from a probability map is not possible. If the component raised an alarm and the comparison of the activity probability map of the anomaly and the reference showed that the probability of using, e.g. the kettle has dropped to 0 the reason was not obvious. One reason could be that the kettle was broken and the new one had not yet been delivered. Another functional related reason could be that the older adult could not use the kettle anymore because he or she could not push the buttons, read the caption, or could not see the pictographs on the buttons. In the first case the older adult would still have been a facilitator (score: + 4) of the kettle and in the second case it would have been a barrier (score: 4). When an alarm is raised, the person in charge must interview the older adult to investigate the reason for the change. Those considerations lead to another important point, the complex effects of changing conditions, e.g. a change in item d450: Walking likely affects other items like d530: Toileting. Our system has the potential to incorporate this complexity in two ways in future work. The first way is to use more refined components. The aim of component 2 was to measure changing body weight as an indicator for malnutrition. Adding important nutrition related information like consumption quantities might add valuable information that allows deeper insights^42^. Component 1 processed IMU data to predict the SPPB and TUG score. The SPPB is comprised of three items and the TUG can be separated into the three items, sit-to-stand, walk, and balance. Since the component only predicted the overall score it was not possible to determine the contribution of each item. A more refined version of the component could predict the subscores of each item to gain more insights. The second way is to use early fusion of the data and middle fusion of the components. An early fusion of the sensor data enables a more accurate estimation of parameters of interest, e.g. the fusion of IMU and motion sensor data allows a precise (i.e. smaller than the Minimal Clinically Important Difference (MCID)) estimation of the gait speed in domestic environments^43^. Since a lot of information is hidden in component 3 and its the activity probability maps a middle fusion with component 1 can give deeper insights in the impairment in daily life caused by reduced mobility. Fusing both components might reveal which part of daily life is affected the most, e.g. a decrease in mobility might lead to less activity on the upper floors of a flat and the resident may not be able to go to the balcony for recreation anymore.

Besides the pre-clinical study and component focused aspects, the usability and the effect on the daily practice of clinicians must be evaluated as well. If the sensitivity is too high many false alarms are raised and that would lead to dwindling acceptance amongst clinicians.

## Conclusion

We introduced the system OdAMS for monitoring the functional status of older adults in daily life. The system linked IMU and home automation sensor data to three different ICF categories and raises an alarm if a change in one category was detected. The application to data of 20 (pre-)frail older adults showed the validity of the system. Moreover, we showed how OdAMS would be used by physiotherapists in two exemplary cases. Combining smart-home sensor systems with the ICF enables clinicians to tailor rehabilitation programmes to individuals and thereby improve their outcomes. Nevertheless, the system could be beneficial for the entire multi-disciplinary team—including physicians, nutritionists, and pharmacists amongst others. Our results are encouraging and future research should be directed to the possible impact on research and society.

OdAMS’s potential impact on research is to enable researchers to conduct high-quality, reproducible, and transparent interdisciplinary research. The ICF provides a common language for describing and classifying a person’s functional abilities across multiple disciplines. Using the ICF categories and items as a basis allows medical professionals from different fields to collaborate and identify relations between different medical aspects. For example, if the component 2 and component 3 are always raising alarms at the same time, that would be an indication for a relation between ICF items b530 weight maintenance functions and e115/e245 products and technology for personal use/time related changes. Regarding interdisciplinary research between physician-scientists and computer scientists, the linking of data to the ICF items bridges the gap between disciplines. Physician–scientists could choose the ICF category and the specific items they want to investigate and what information they need. Based on that decision they can involve computer scientists to choose the data and the sensors according to their needs.

In society, the system has the potential to improve the quality of life of older adults by enabling them to living longer independently and to meet their psychological needs. In addition, the system enables physicians and medical professionals to apply individualised care and rehabilitation interventions. This would improve the well-being and physical condition of older people. It also supports the goal of rehabilitation to enable older people to maintain their independent daily lives. Therefore, the system supports physicians at assessing and keeping track of functional abilities and the effect of exercise programmes.

## Supplementary Information

Supplementary Information.

Supplementary Information.

Supplementary Information.

## Data Availability

All data generated or analysed during this study are included in this published article.

## Code availability

The source code is available in the supplementary materials for academic purposes.
