# Supplementary figures and images for: A system for monitoring the functional status of older adults in daily life
Source: Sci Rep. 2023 Jul 31;13:12396. doi: 10.1038/s41598-023-39483-x (PMC10390547; doi:10.1038/s41598-023-39483-x)

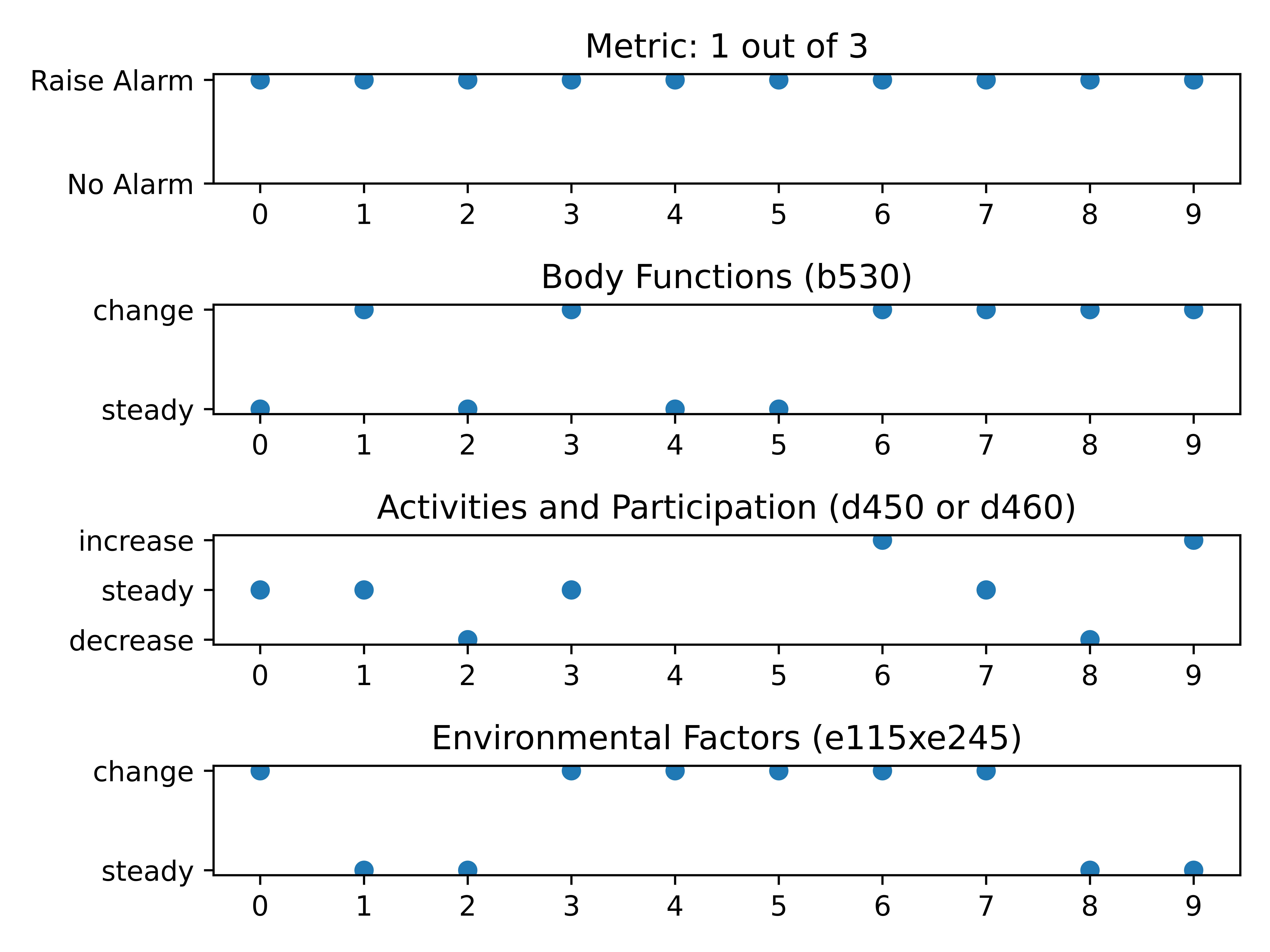

Supplement: Supplementary file 1 — Supplementary Information. [file 41598_2023_39483_MOESM1_ESM.zip › sourcecode/figures/Fig.S1.png]

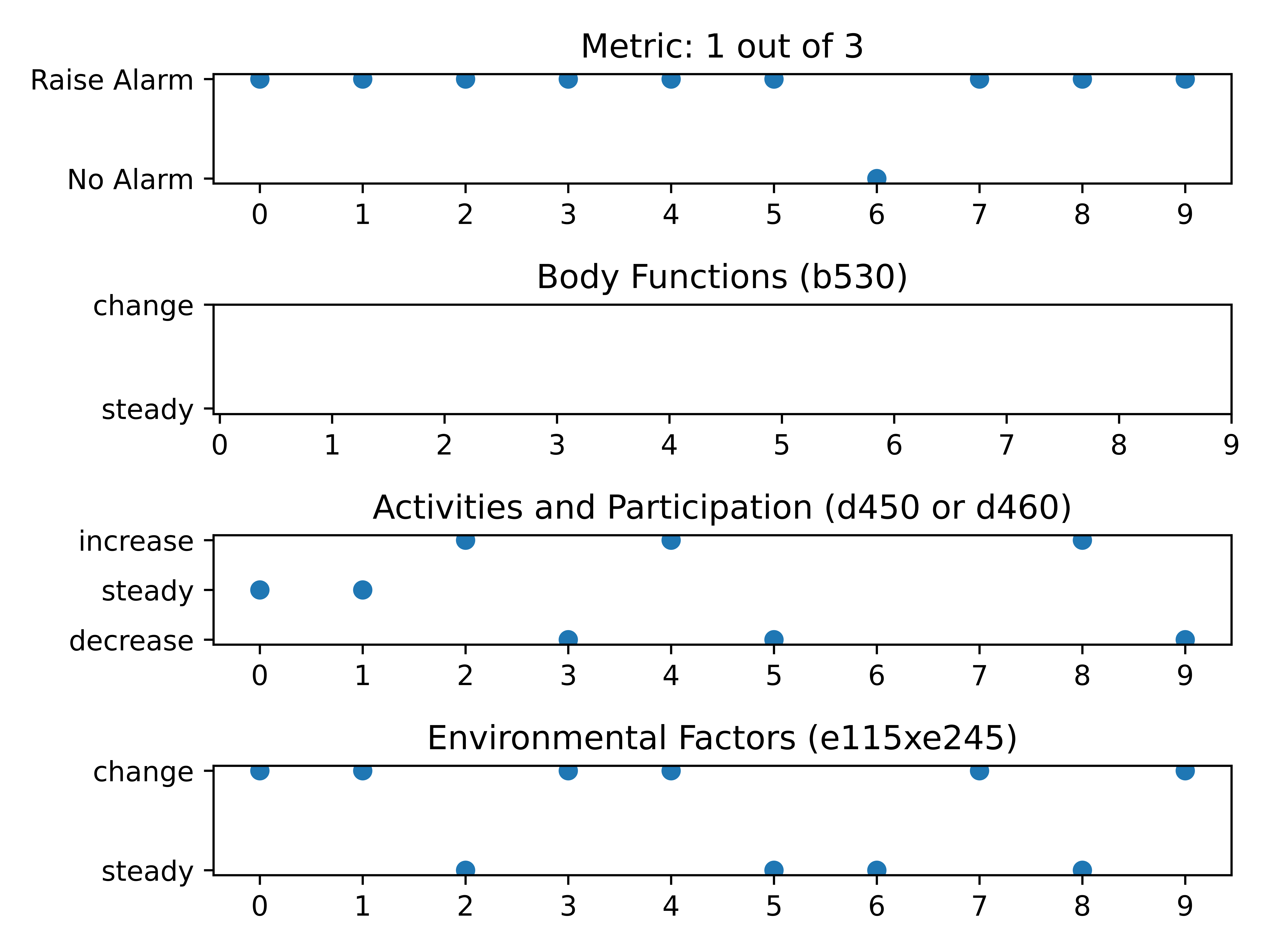

Supplement: Supplementary file 1 — Supplementary Information. [file 41598_2023_39483_MOESM1_ESM.zip › sourcecode/figures/Fig.S10.png]

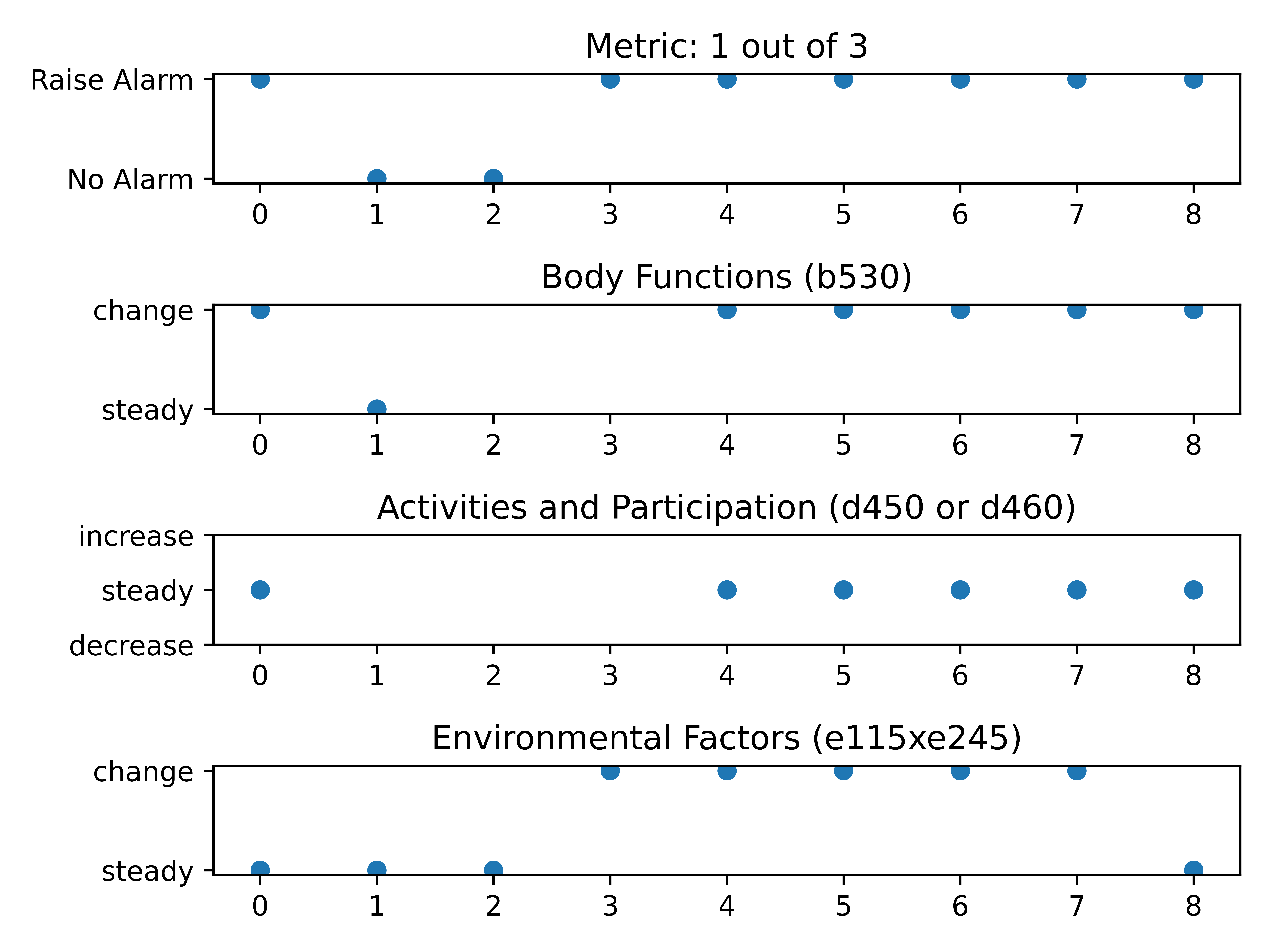

Supplement: Supplementary file 1 — Supplementary Information. [file 41598_2023_39483_MOESM1_ESM.zip › sourcecode/figures/Fig.S11.png]

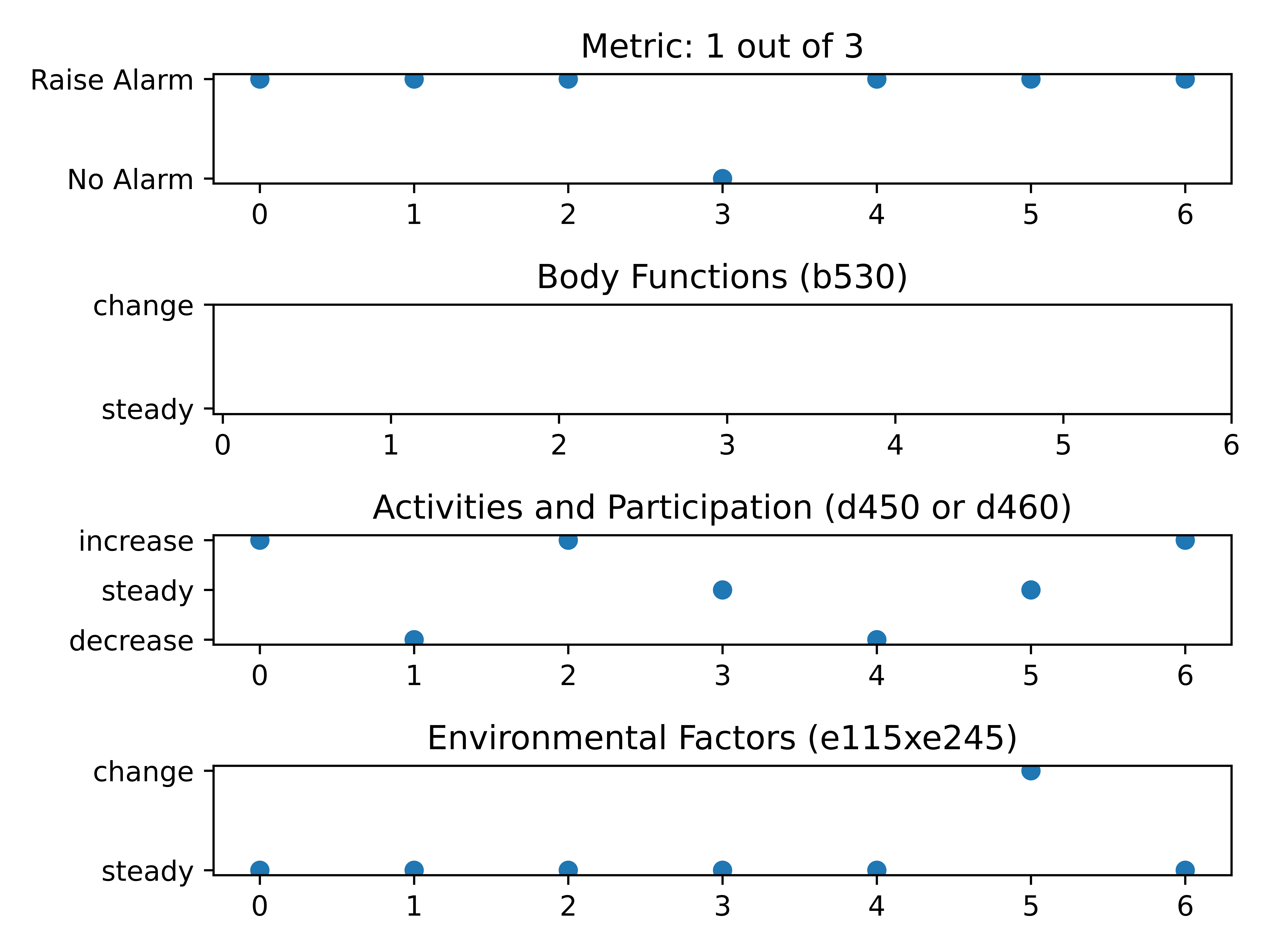

Supplement: Supplementary file 1 — Supplementary Information. [file 41598_2023_39483_MOESM1_ESM.zip › sourcecode/figures/Fig.S12.png]

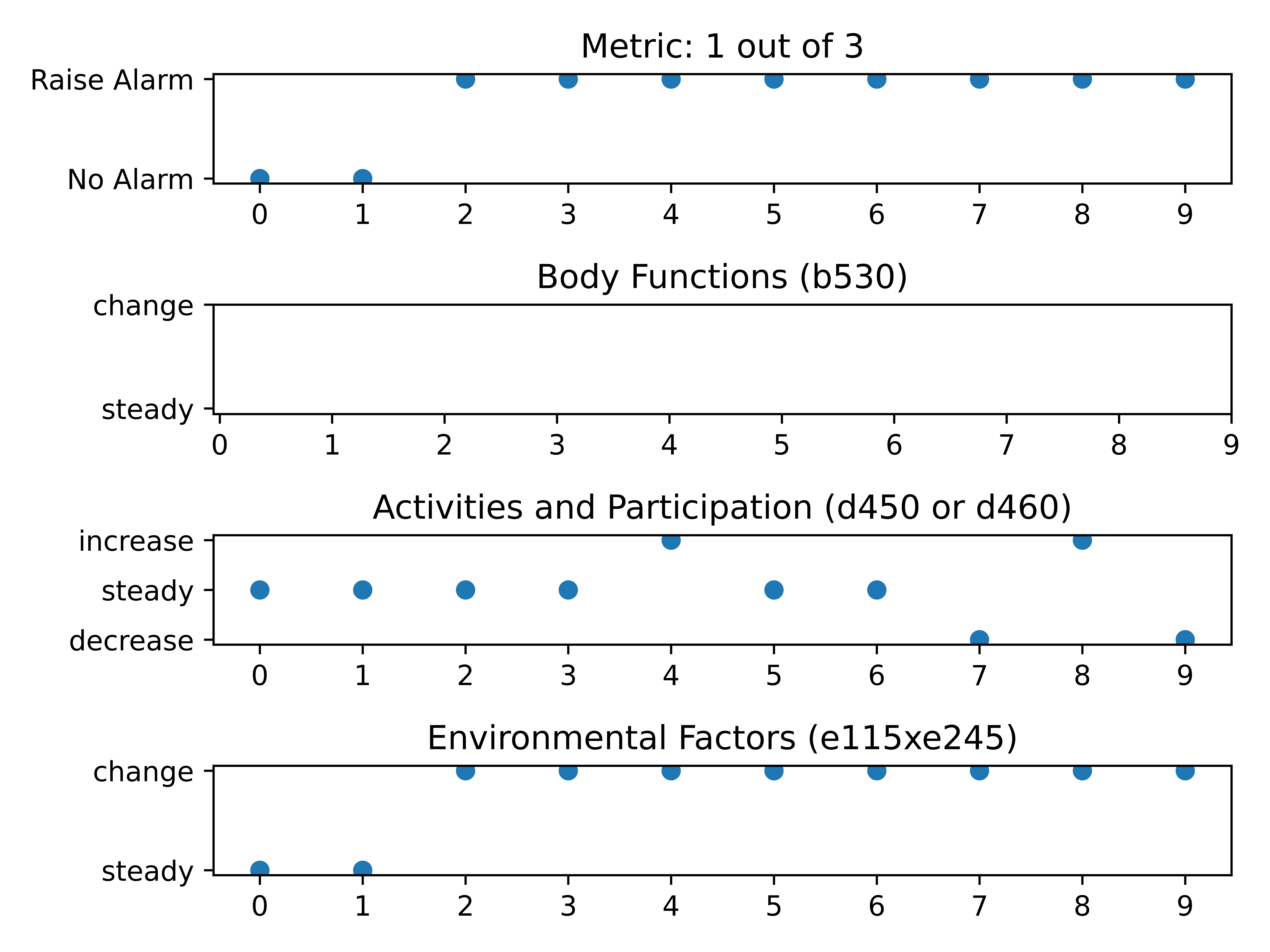

Supplement: Supplementary file 1 — Supplementary Information. [file 41598_2023_39483_MOESM1_ESM.zip › sourcecode/figures/Fig.S13.png]

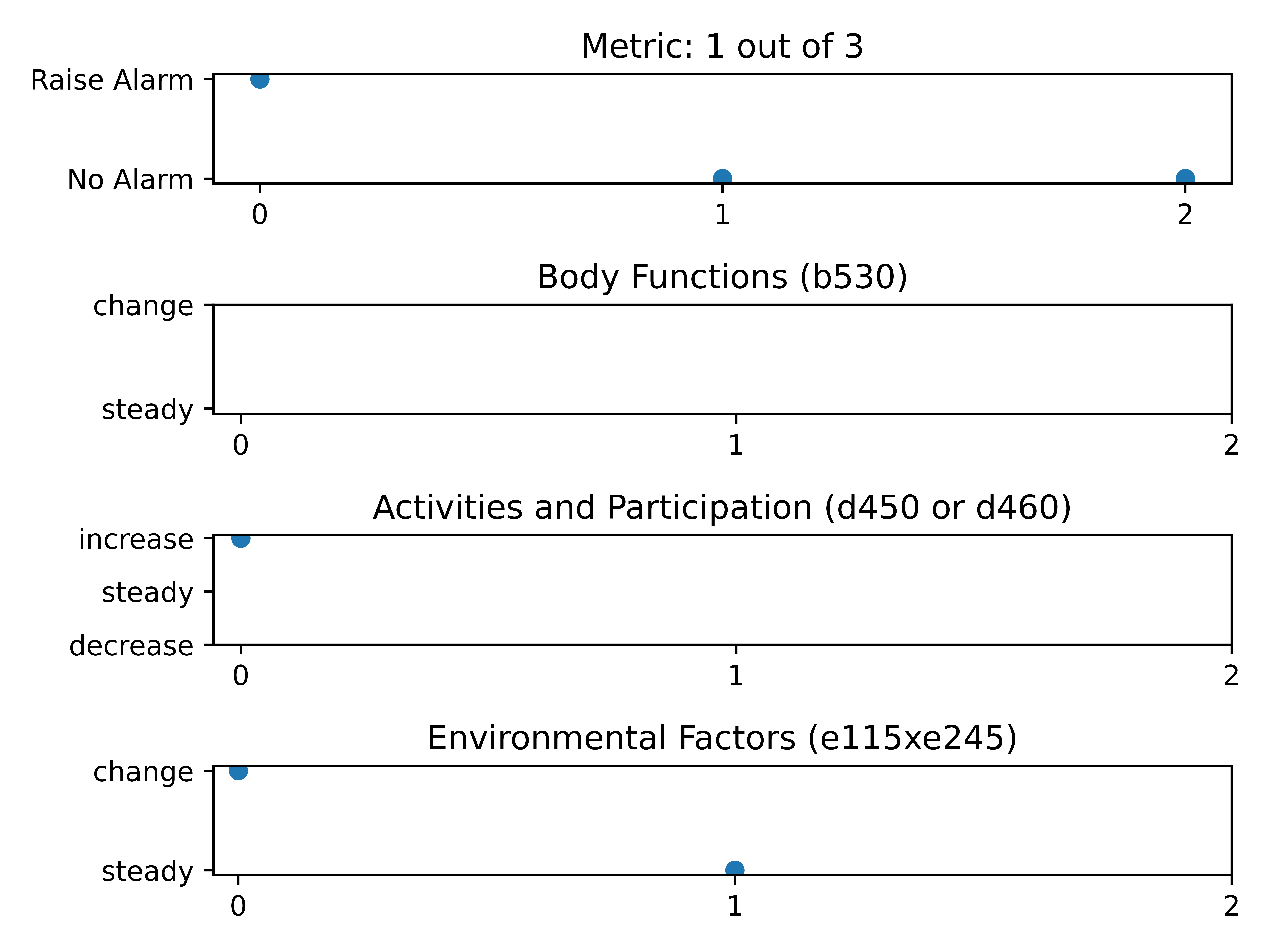

Supplement: Supplementary file 1 — Supplementary Information. [file 41598_2023_39483_MOESM1_ESM.zip › sourcecode/figures/Fig.S14.png]

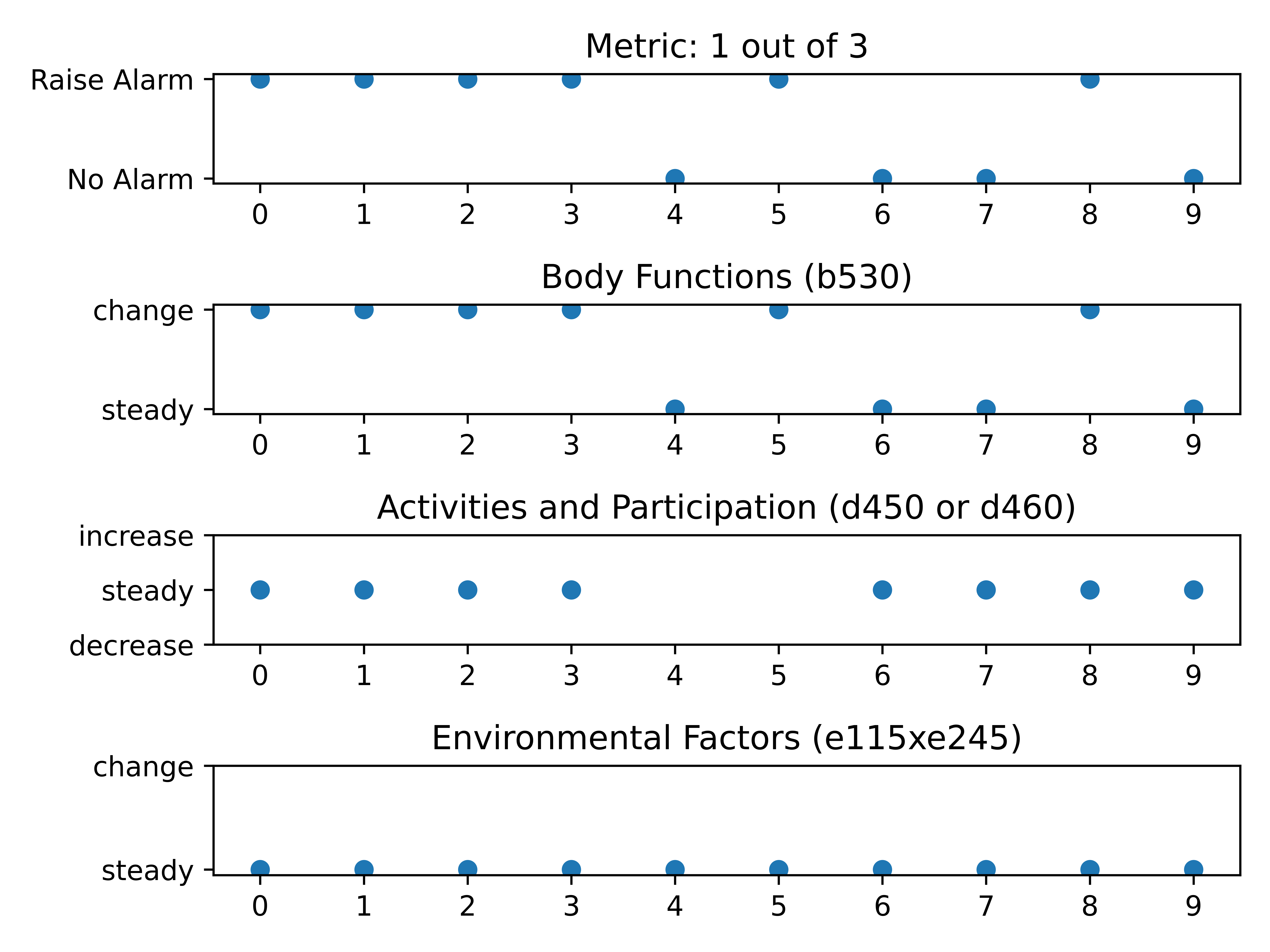

Supplement: Supplementary file 1 — Supplementary Information. [file 41598_2023_39483_MOESM1_ESM.zip › sourcecode/figures/Fig.S15.png]

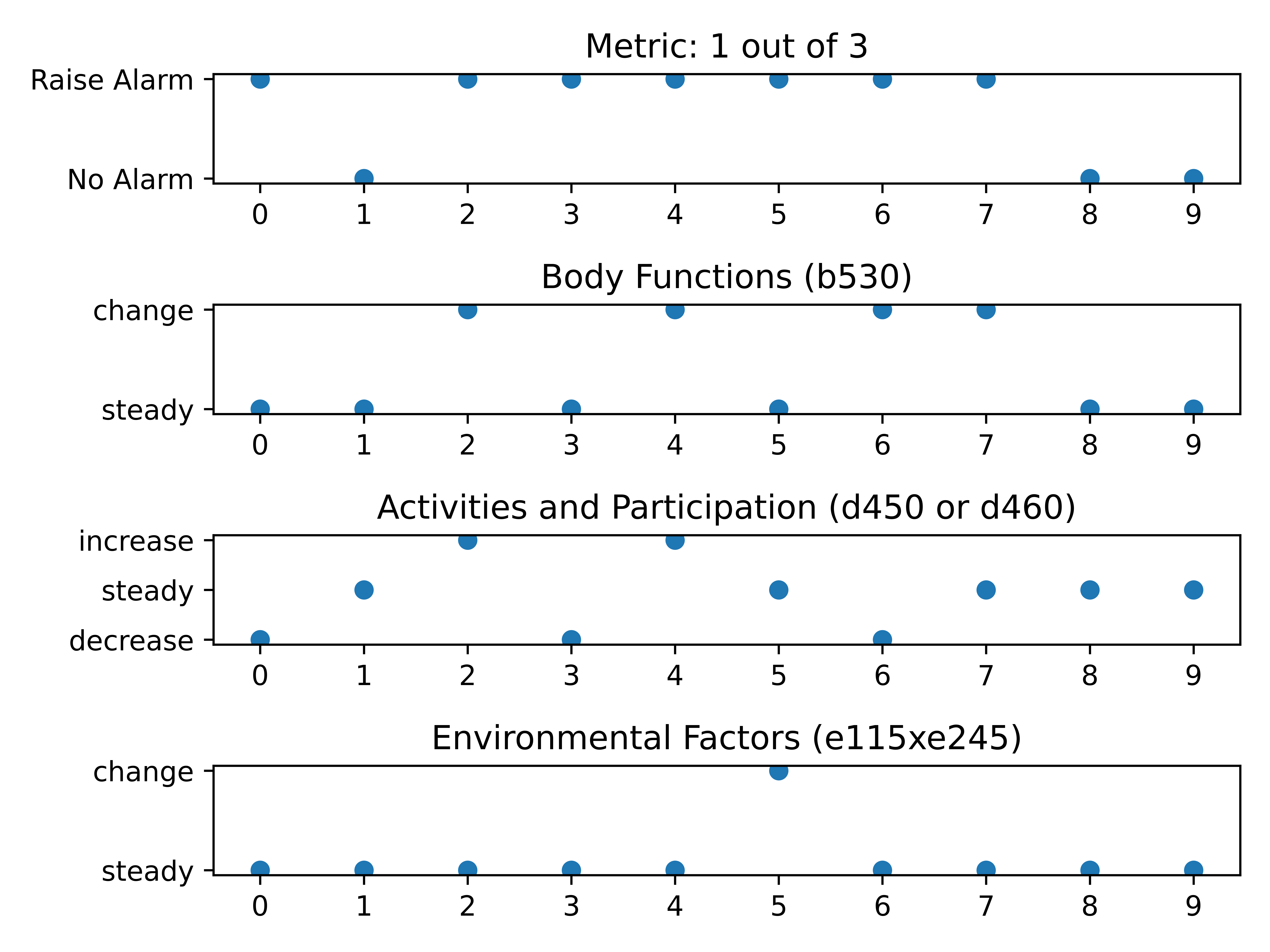

Supplement: Supplementary file 1 — Supplementary Information. [file 41598_2023_39483_MOESM1_ESM.zip › sourcecode/figures/Fig.S16.png]

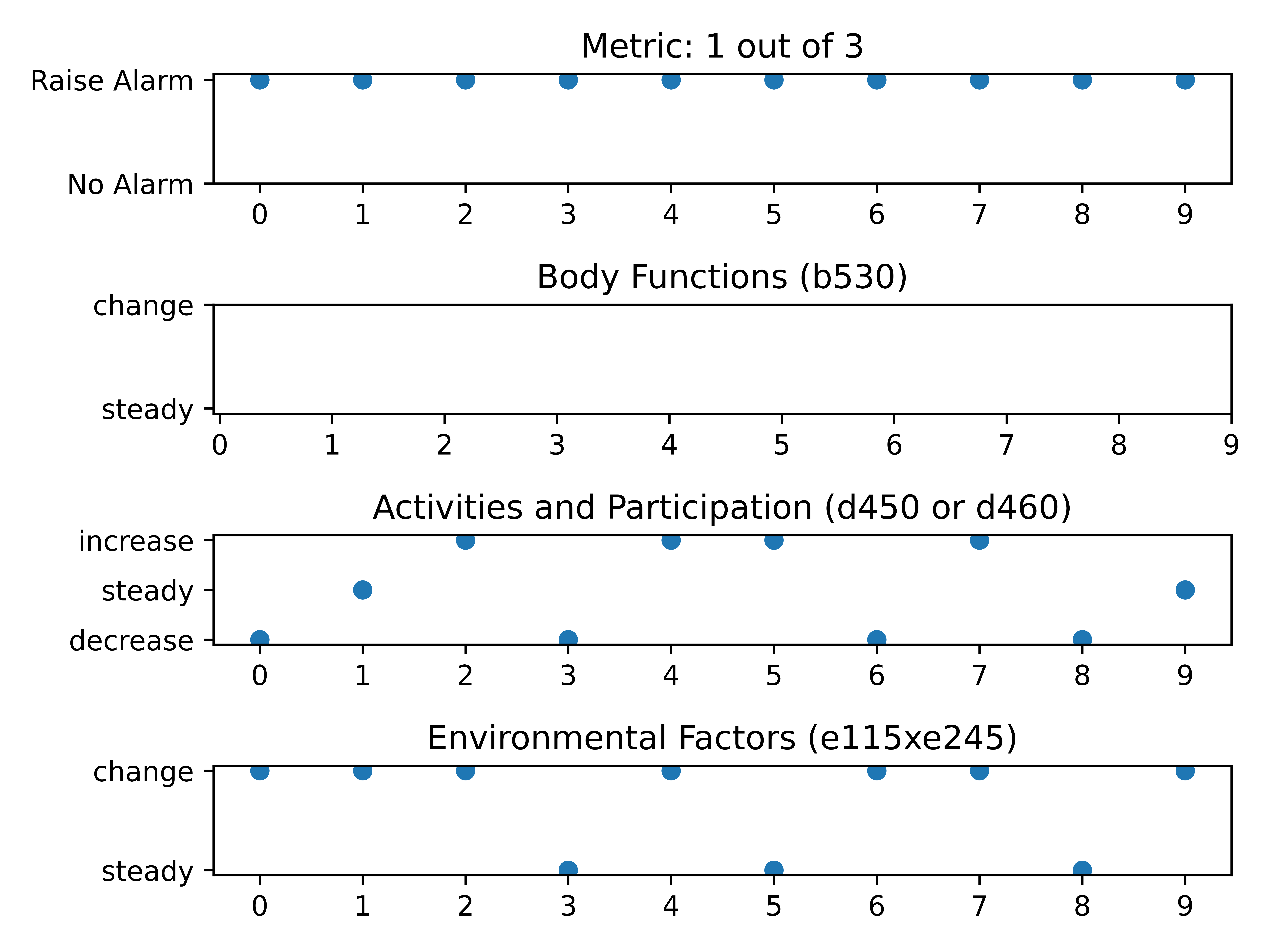

Supplement: Supplementary file 1 — Supplementary Information. [file 41598_2023_39483_MOESM1_ESM.zip › sourcecode/figures/Fig.S17.png]

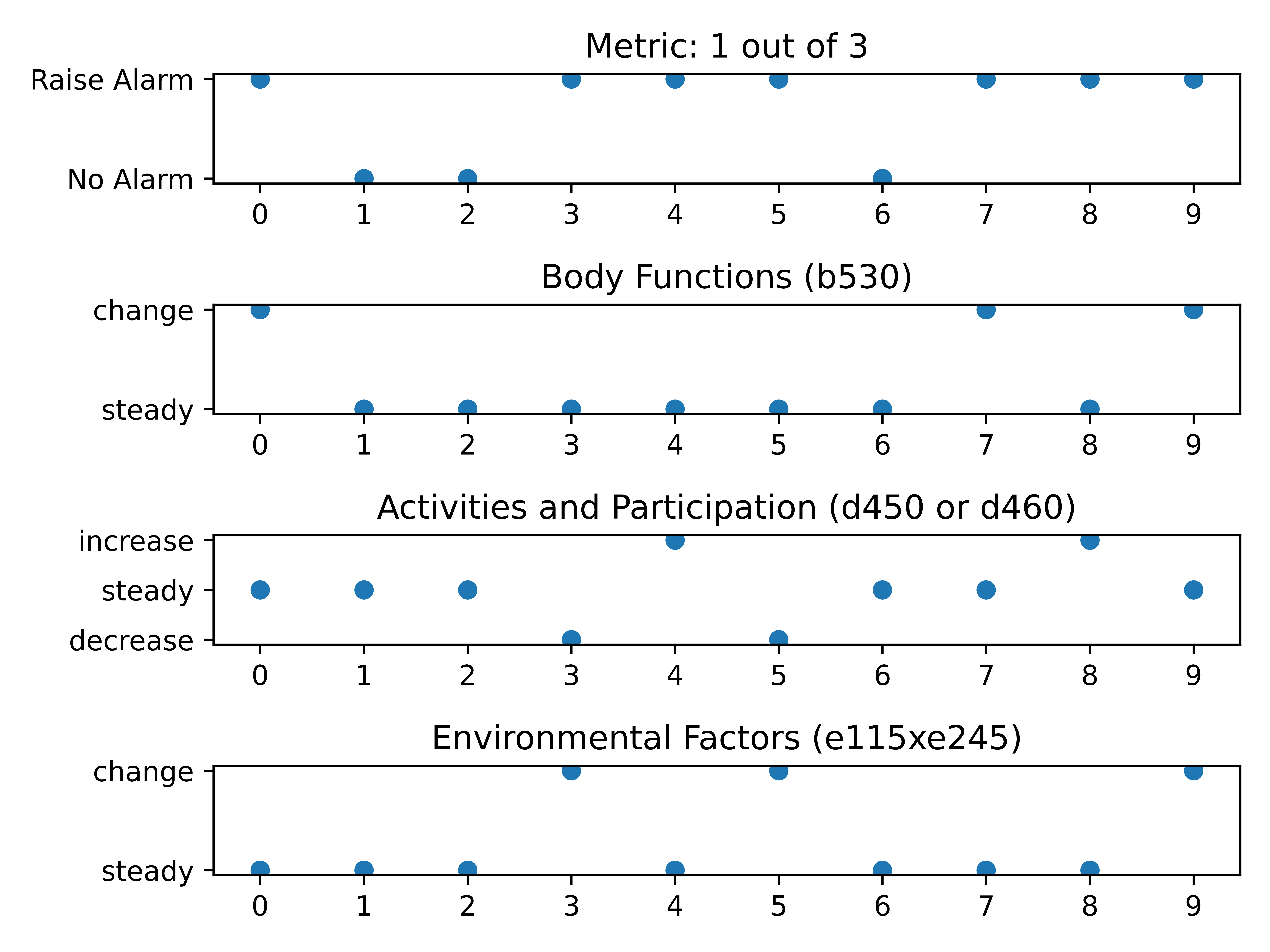

Supplement: Supplementary file 1 — Supplementary Information. [file 41598_2023_39483_MOESM1_ESM.zip › sourcecode/figures/Fig.S18.png]

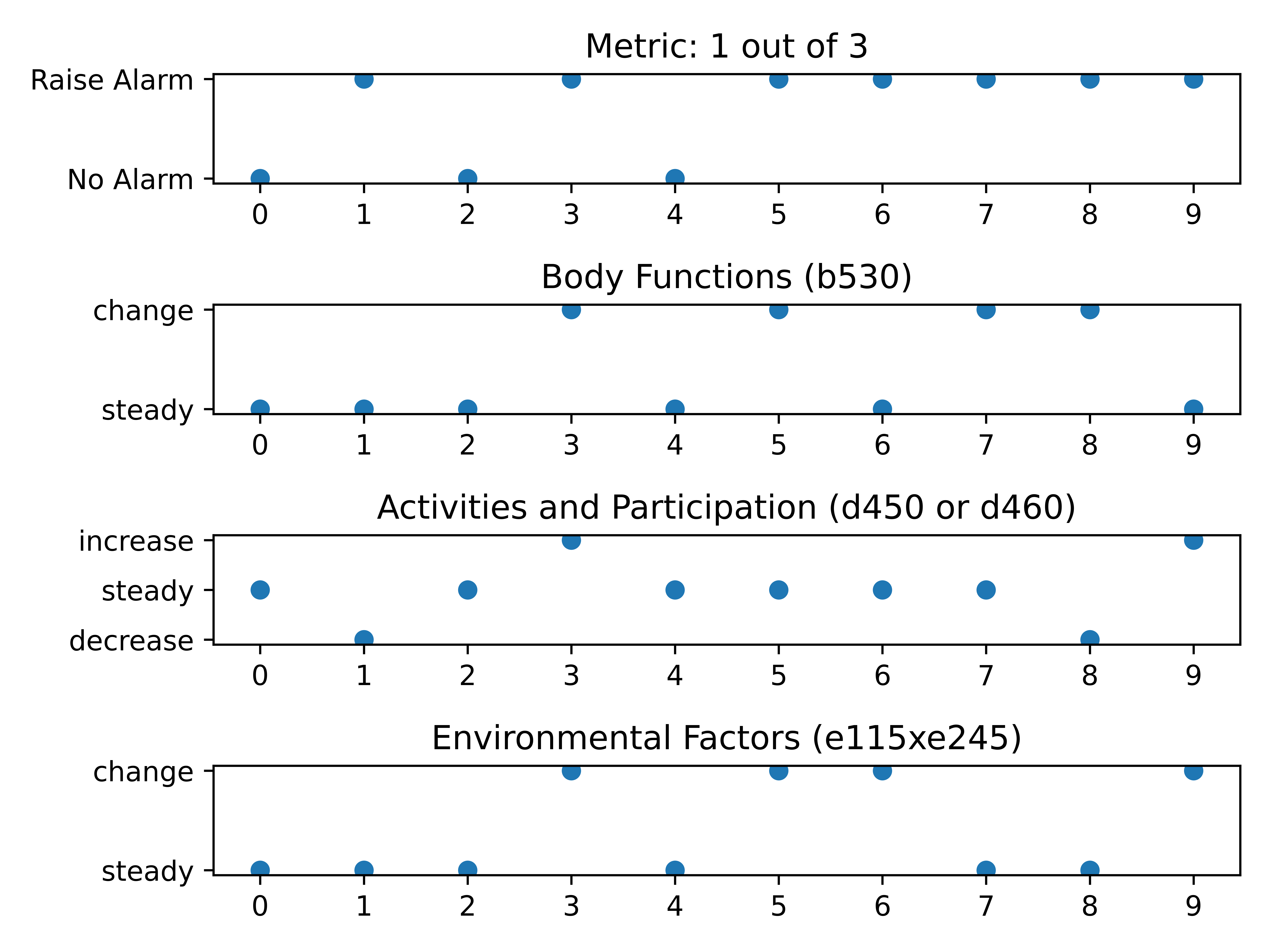

Supplement: Supplementary file 1 — Supplementary Information. [file 41598_2023_39483_MOESM1_ESM.zip › sourcecode/figures/Fig.S2.png]

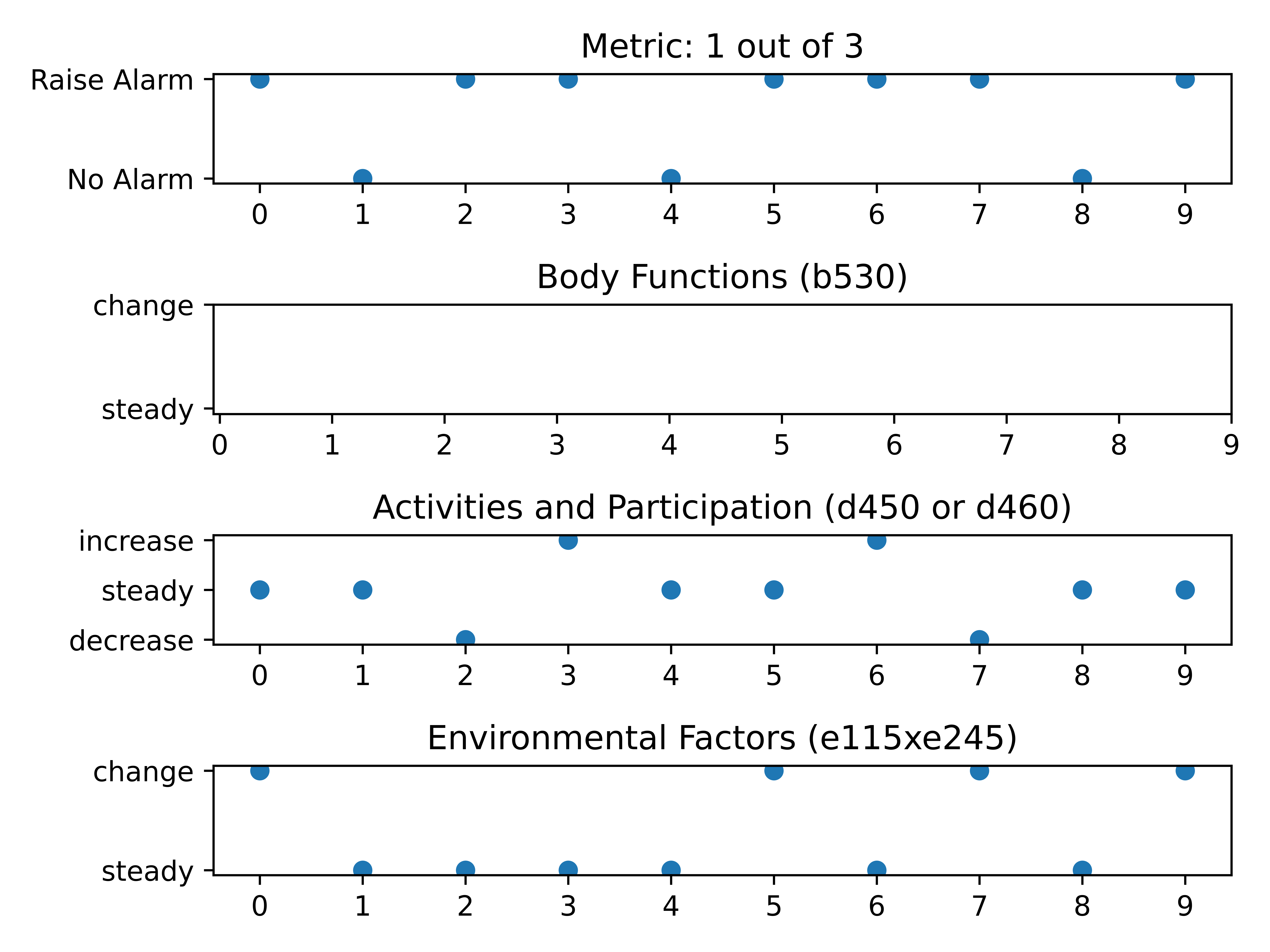

Supplement: Supplementary file 1 — Supplementary Information. [file 41598_2023_39483_MOESM1_ESM.zip › sourcecode/figures/Fig.S3.png]

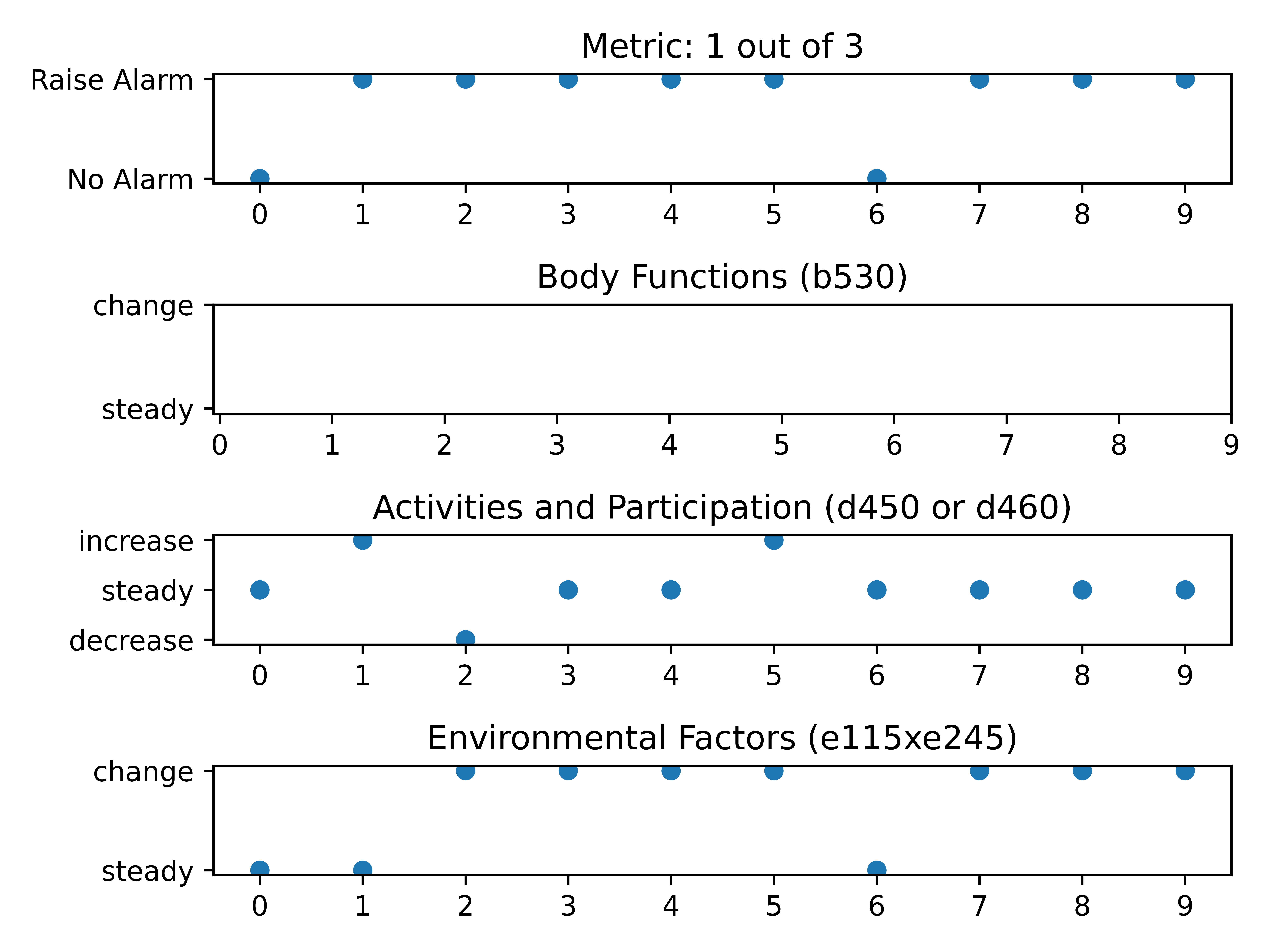

Supplement: Supplementary file 1 — Supplementary Information. [file 41598_2023_39483_MOESM1_ESM.zip › sourcecode/figures/Fig.S4.png]

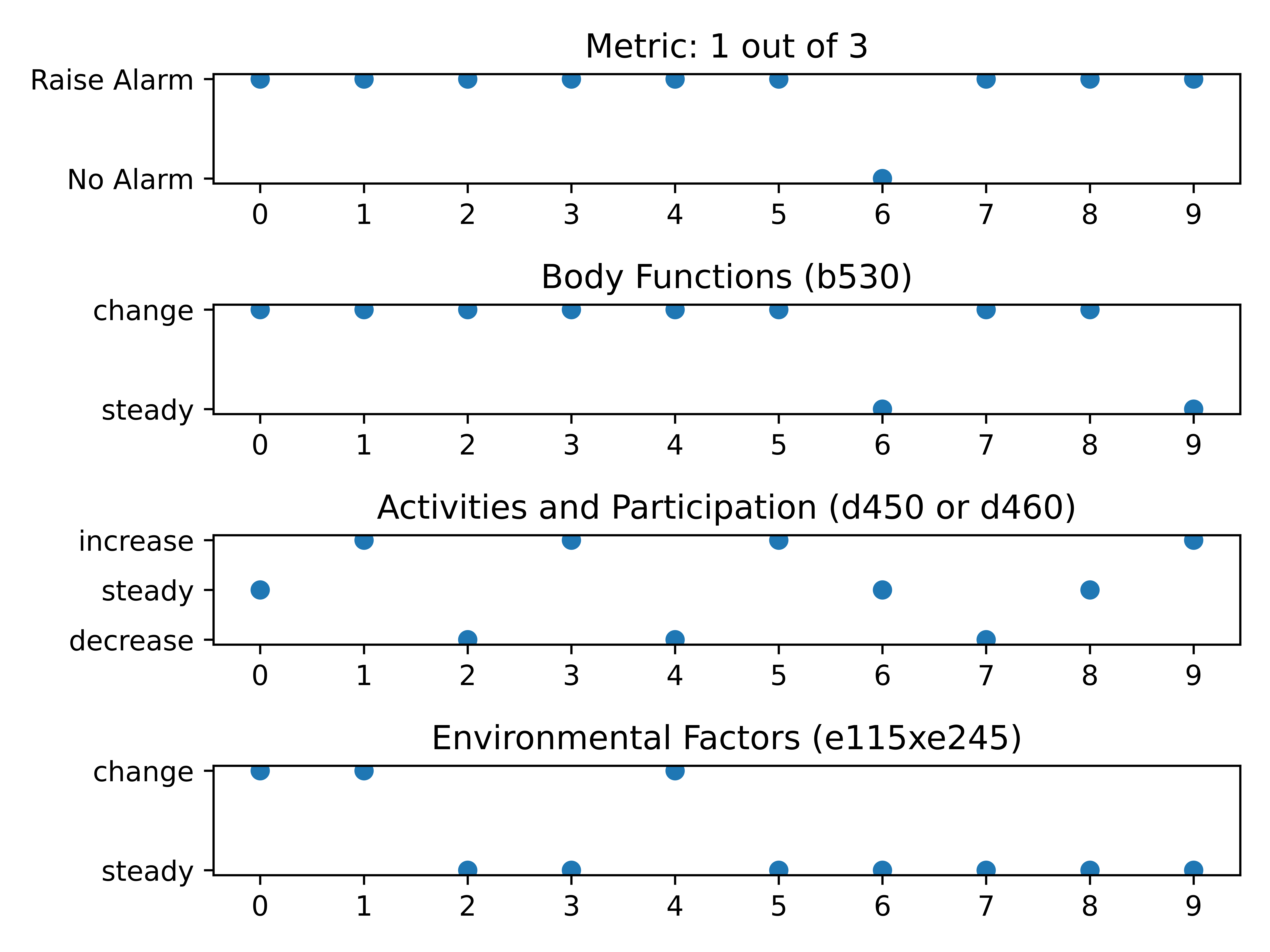

Supplement: Supplementary file 1 — Supplementary Information. [file 41598_2023_39483_MOESM1_ESM.zip › sourcecode/figures/Fig.S5.png]

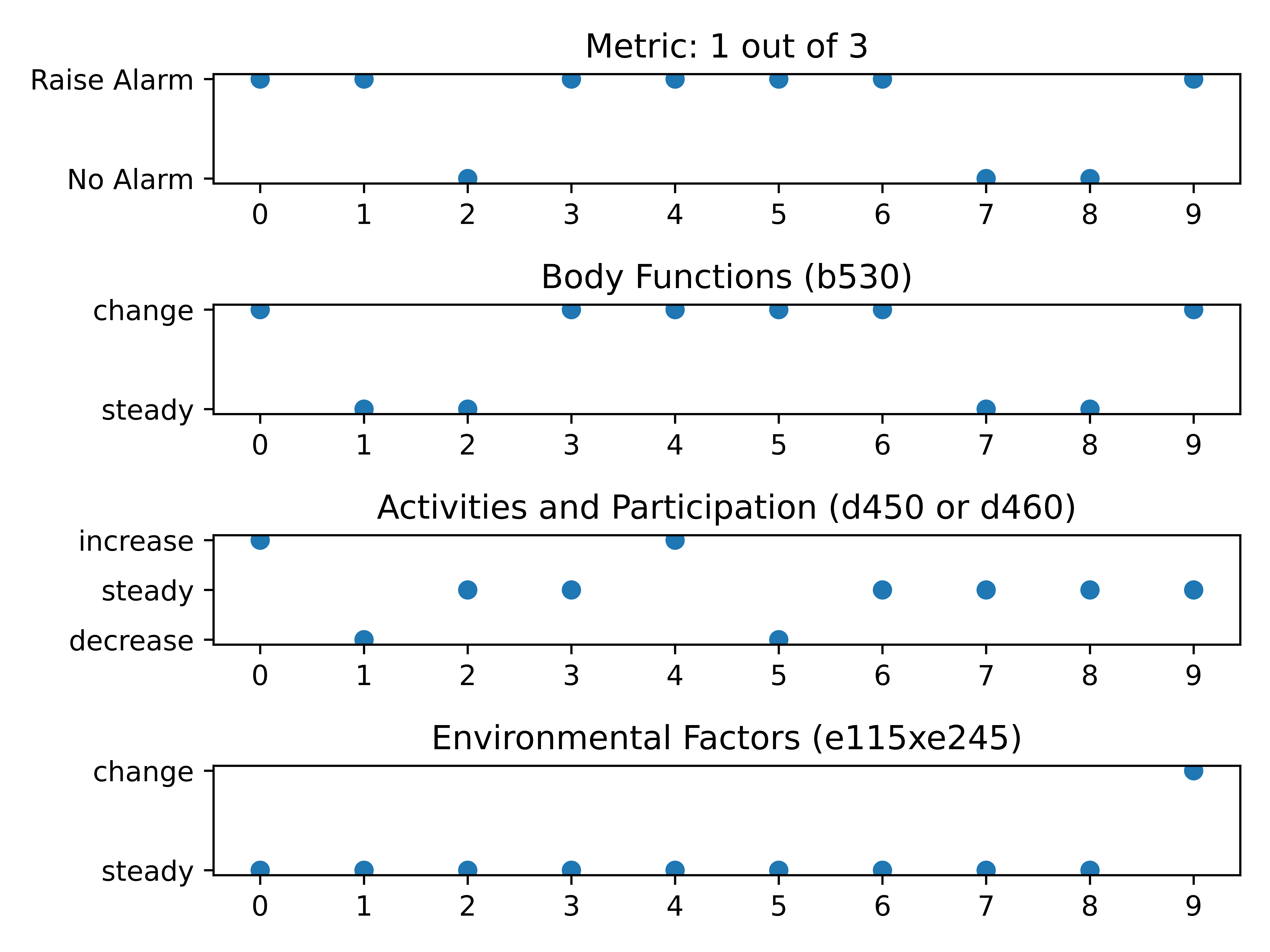

Supplement: Supplementary file 1 — Supplementary Information. [file 41598_2023_39483_MOESM1_ESM.zip › sourcecode/figures/Fig.S6.png]

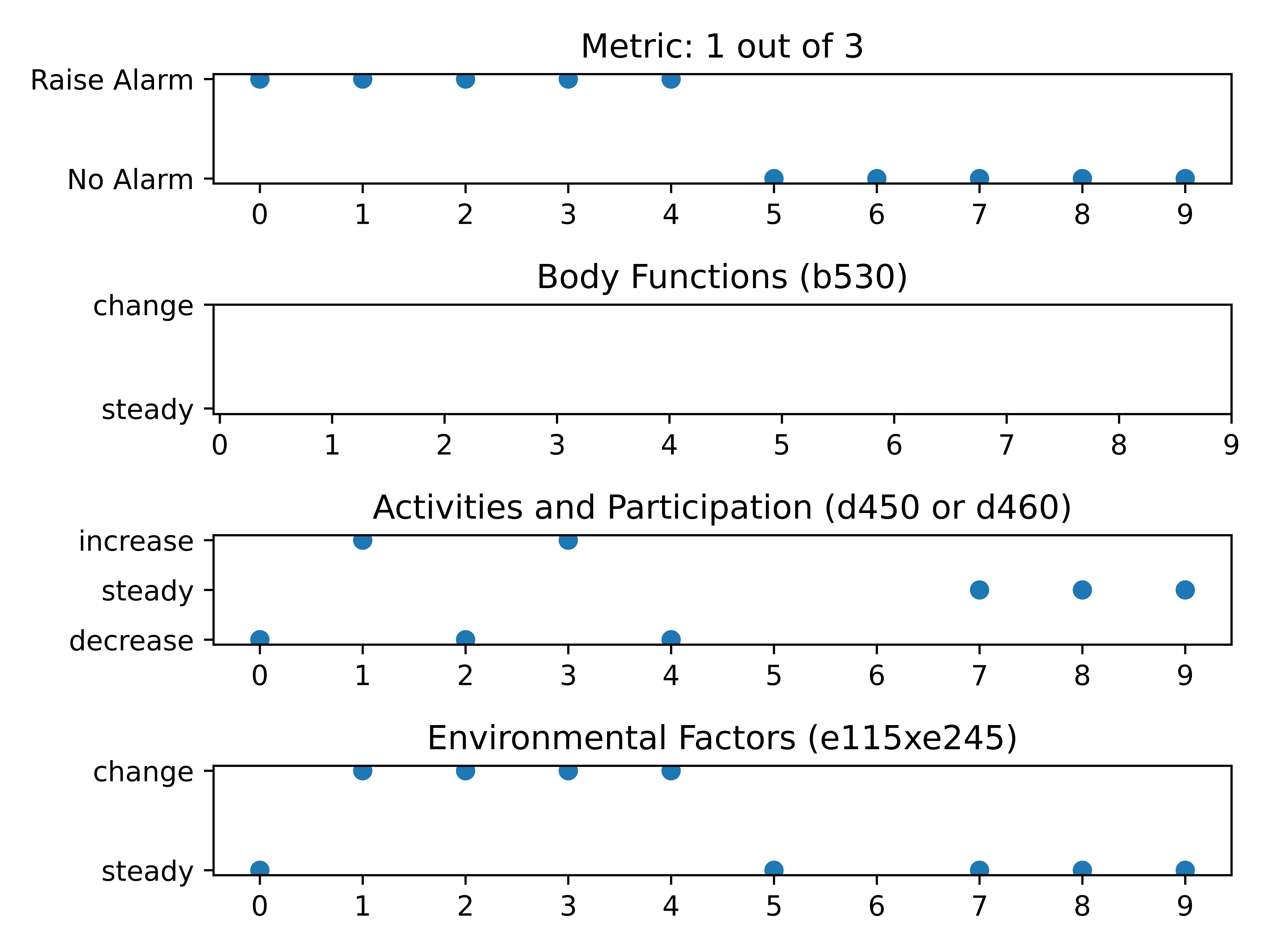

Supplement: Supplementary file 1 — Supplementary Information. [file 41598_2023_39483_MOESM1_ESM.zip › sourcecode/figures/Fig.S7.png]

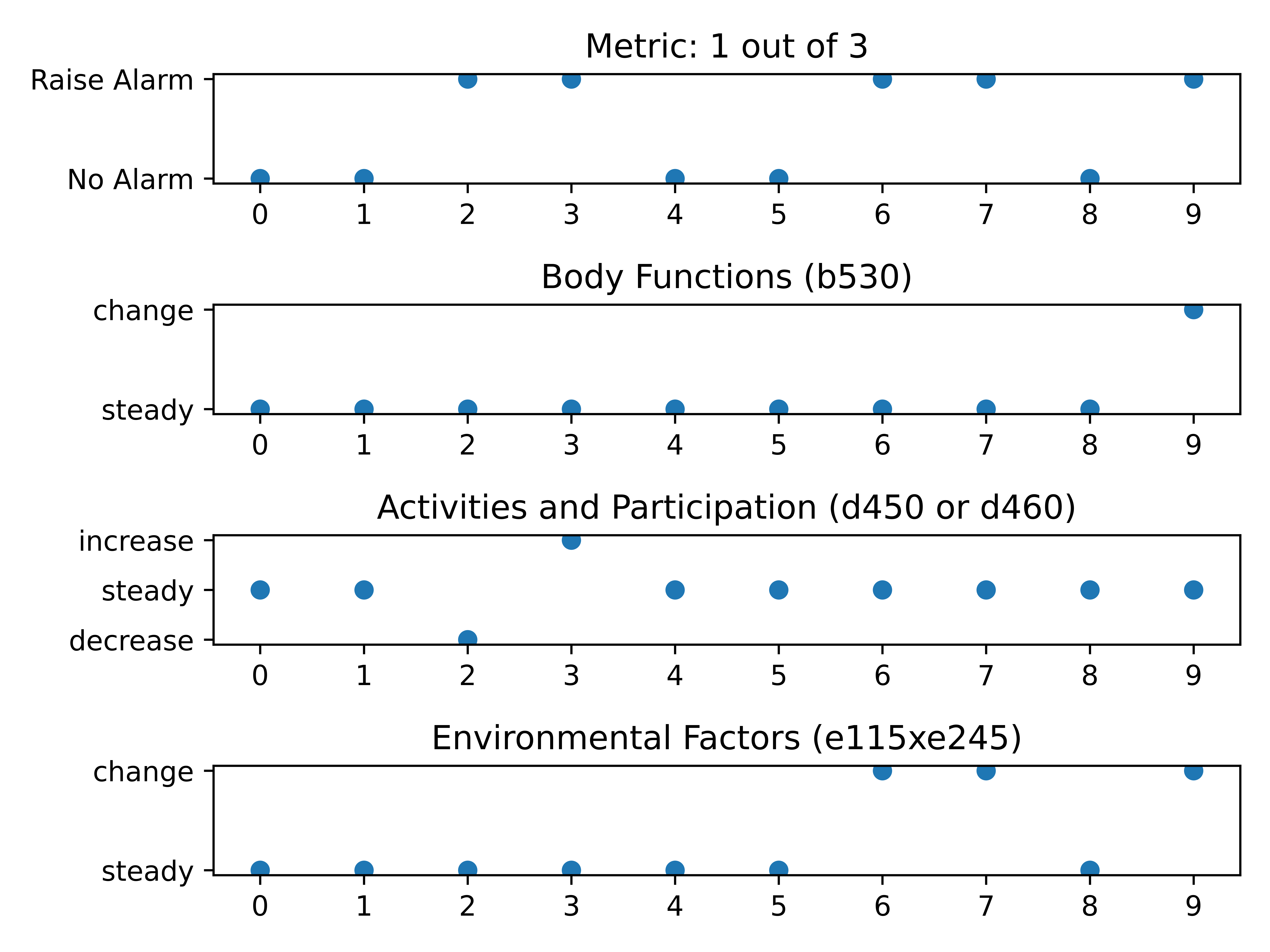

Supplement: Supplementary file 1 — Supplementary Information. [file 41598_2023_39483_MOESM1_ESM.zip › sourcecode/figures/Fig.S8.png]

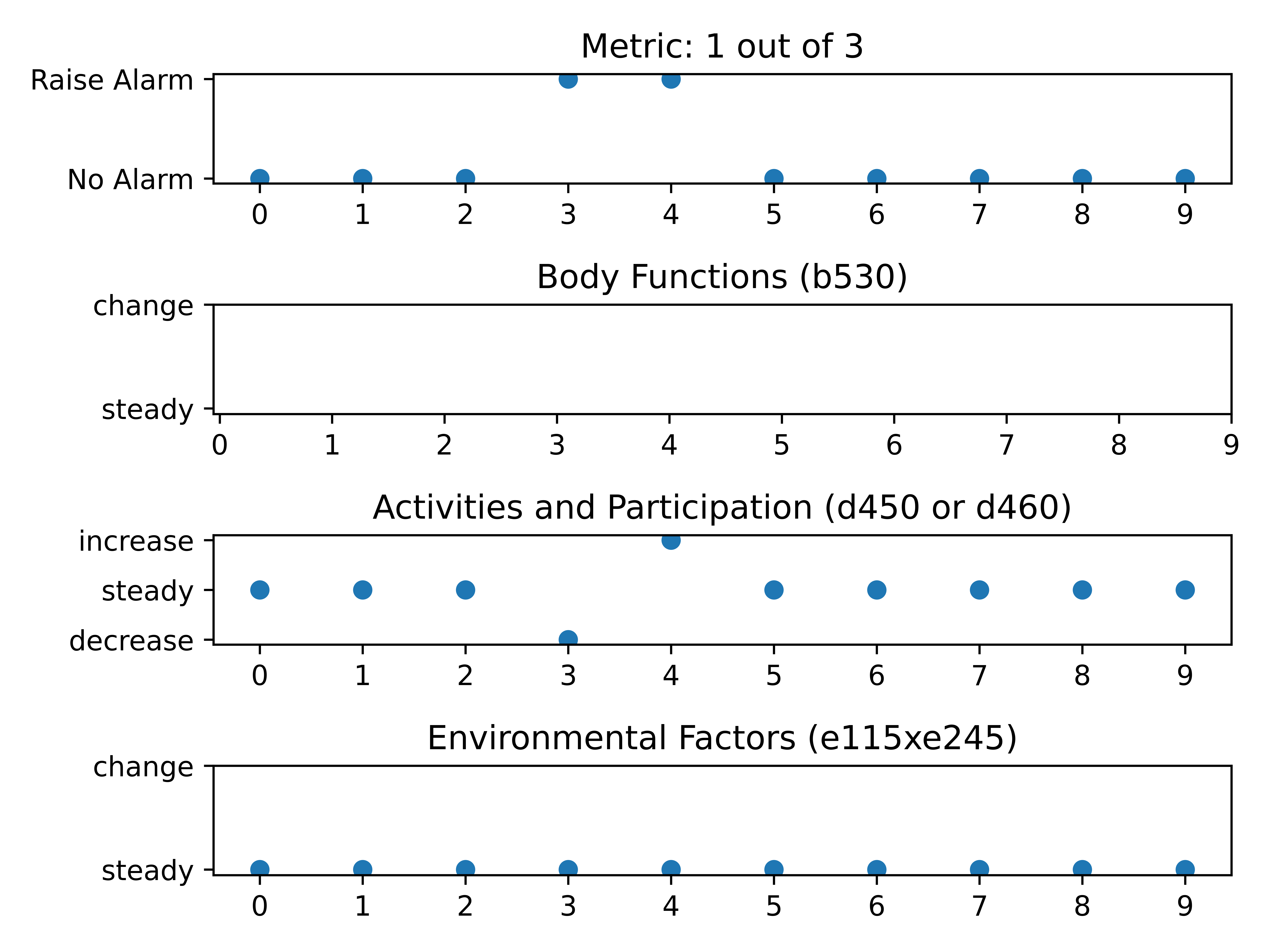

Supplement: Supplementary file 1 — Supplementary Information. [file 41598_2023_39483_MOESM1_ESM.zip › sourcecode/figures/Fig.S9.png]

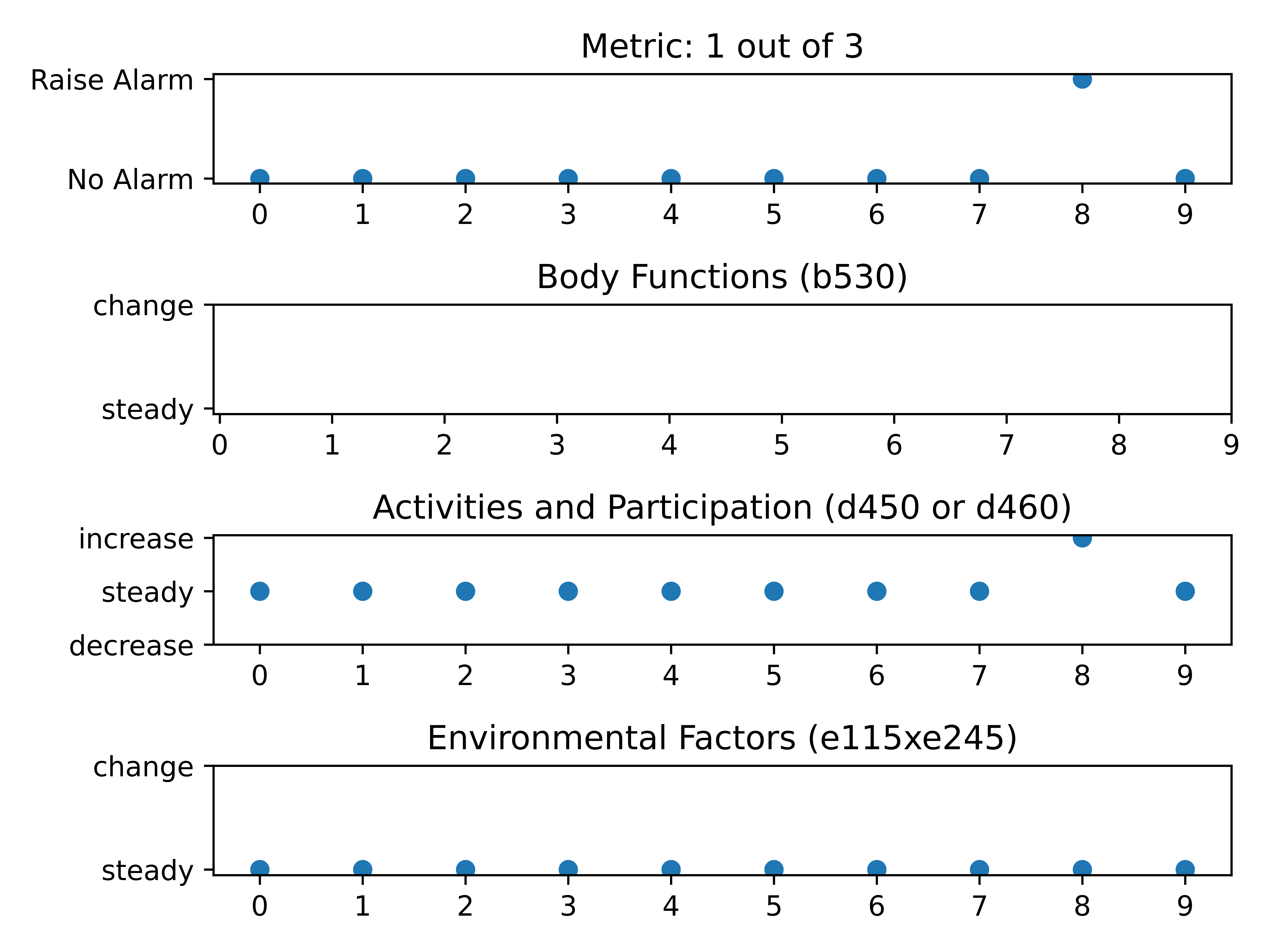

Supplement: Supplementary file 1 — Supplementary Information. [file 41598_2023_39483_MOESM1_ESM.zip › sourcecode/figures/Fig.Smueller.png]

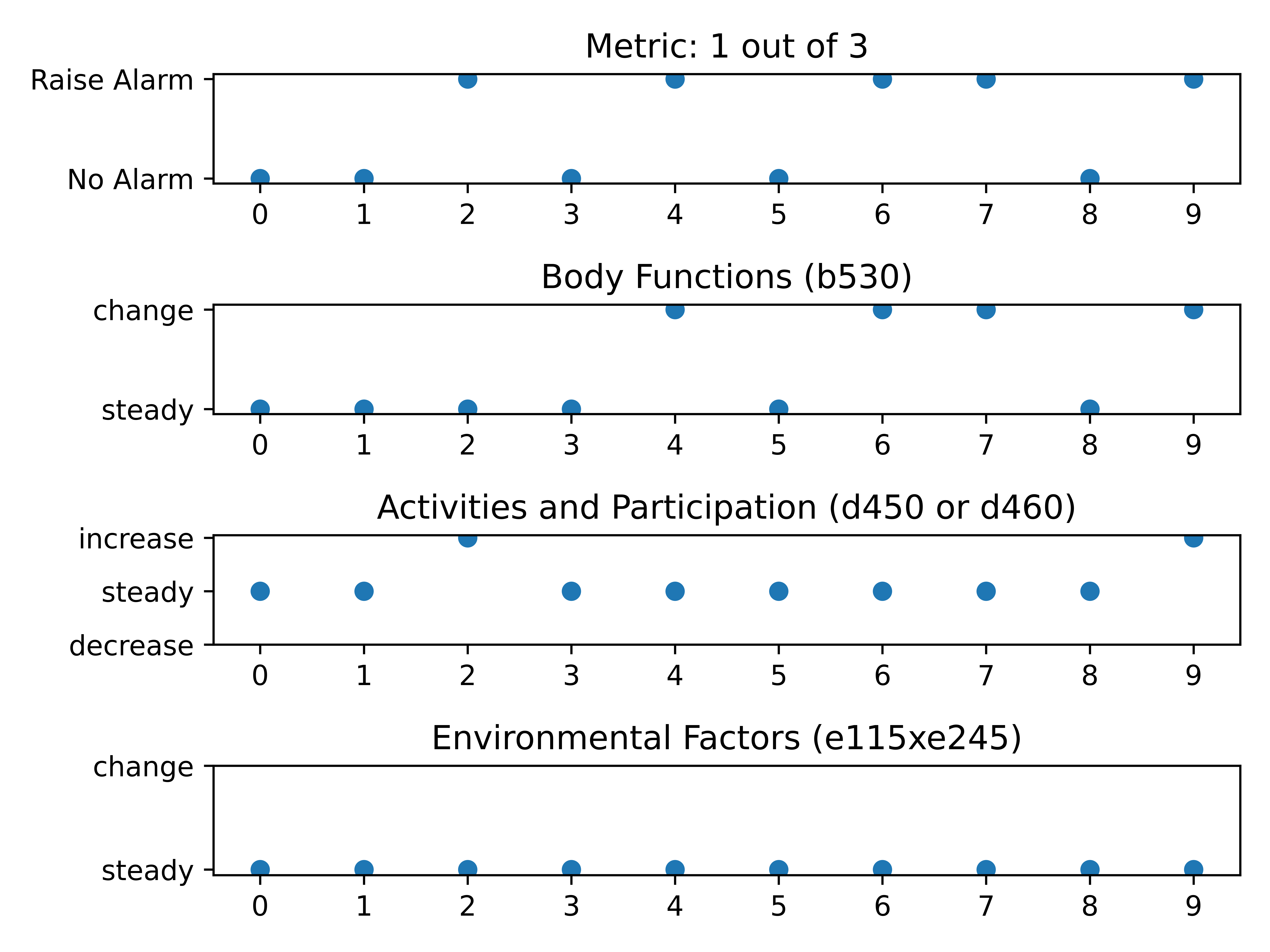

Supplement: Supplementary file 1 — Supplementary Information. [file 41598_2023_39483_MOESM1_ESM.zip › sourcecode/figures/Fig.Swinter.png]
